# A Tale of Two Tissues: *AtGH9C1* Is an Endo-β-1,4-Glucanase Involved in Root Hair and Endosperm Development in *Arabidopsis*

**DOI:** 10.1371/journal.pone.0049363

**Published:** 2012-11-16

**Authors:** Elena del Campillo, Sivacharan Gaddam, Dorinne Mettle-Amuah, Jean Heneks

**Affiliations:** Department of Cell Biology and Molecular Genetics, University of Maryland, College Park, Maryland, United States of America; Lawrence Berkeley National Laboratory, United States of America

## Abstract

Arabidopsis AtGH9C1 is an endo-β-1,4-glucanase possessing a carbohydrate-binding domain (CBM49). Analysis of *AtGH9C1* expression by promoter-reporter GUS, RT-PCR, public transcriptome databases and GFP protein tagging demonstrated a high and selective expression of *AtGH9C1* in root hairs and in the endosperm. Expression in root hair cells started prior to bulge formation and continued during hair elongation. *AtGH9C1* expression increased with treatments that increase density (ACC) or length (sucrose) of root hairs. Expression in the endosperm extended sequentially to the micropylar, peripheral and chalazal compartments. A mutant with reduced *AtGH9C1* expression had a delay in germination and a marked reduction in root hair presence. Complementation of the mutant partially improved both germination and root hair density. Experiments with ectopically expressed AtGH9C1-GFP with and without the CBM49, demonstrated that both forms of the protein are secreted and that CBM49 targets the protein to specific regions of the cell wall, but what makes these regions special is still unknown. The amino acid alignment of angiosperm GH9 genes with C-terminal extensions illustrate that AtGH9C1 belongs to a different clade than its tomato homolog, S1GH9C1. The latter has a CBM49 that was shown to bind crystalline cellulose. We suggest that AtGH9C1 is associated with the weakening of the cell wall during formation and growth of the root hair as well as with the sequential anterior-posterior breakdown of the endosperm cell wall that provides space for the growing embryo. Thus, is likely that the CBM49 of AtGH9C1 recognizes a form of cellulose or glucan polymer that is prevalent in the wall of these specialized tissues and that is different than the one recognized by S1GH9C1.

## Introduction

The role of hydrolytic (β-1, 4) glucanases in plant cell wall loosening is not clear albeit these enzymes were formerly envisioned as the prime movers in the wall expansion process [1], [2]. All plants have genes that encode endo (β-1, 4) –glucanases, EC 3.2.1.4, that catalyze the breaking of β-1, 4 glycosidic bonds in the interior of longer β-1, 4 glucose chains. While it is not known if they can break the glycosidic bonds of the crystalline cellulose matrix [3], [4], it is recognized that this activity could contribute to the loosening of the cell wall to make it more pliable for expansion, [3], [5]. The family of (β-1, 4) glucanases belongs to the Glycosyl Hydrolase family 9 or GH9 (CAZy; http://www.cazy.org). This family is a member of the clan CL0059 (http://pfam.sanger.ac.uk/clan/CL0059), enzymes that have a three dimensional structure composed of 6 helical hairpins.

The GH9 family in plants is extended, divergent and is subdivided into classes A, B and C, all having the conserved catalytic module (∼450AA) composed of two signatures that distinguish the GH9 endoglucanases from other hydrolases [6], [7]. The GH9 class C is of special interest because it comprises putative secreted proteins of ∼600AA containing an amino acid extension at the C-terminus (∼100 -amino acids long) that constitute a carbohydrate binding module (CBM) [8]. A CBM is a short amino-acid sequence that folds into a discrete three-dimensional structure forming a putative carbohydrate binding cleft. Most prokaryote enzymes that hydrolyze insoluble polysaccharides (i.e. cellulases, amylases, and chitinases) have a CBM in addition to a catalytic module to facilitate hydrolysis. The CBM in bacteria cellulases is important [9], because it helps position the enzyme’s catalytic site on the microfibrils for efficient breakage of the endoglycosidic bonds [10], [11]. In some cases, associating a different CBM to the same catalytic module conferred binding specificity to different cellulose forms (amorphous vs. crystalline), [12]. Crystalline cellulose is found at the core of all cellulose microfibrils, but at the surface of the microfibril there are different percentage of amorphous cellulose depending on the size of the microfibrils [13]. The thicker microfibrils found in secondary walls have lower surface to volume ratio and only 6∼7% amorphous cellulose, while the smaller microfibrils found in primary cell wall have 33-80% amorphous cellulose [13], [14].

The CBM of plant GH9 class C constitutes a distinctive family, which has been given the designation of CBM49 (CAZy). The CBM49 of S1GH9C1, (SlCel9C1), an endo (β-1, 4) glucanase from *Solanum lycopersicum*, was characterized first and shown to bind crystalline cellulose *in vitro* [15]. Thus, it was suggested that S1GH9C1, might facilitate cellulose degradation during tomato ripening [15]. However, the physiological role of S1GH9C1, and for that matter the role of all plant (GH9) class C enzymes, remains unknown.

We selected *A. thaliana* to analyze the role of one GH9 gene containing a CBM49 referred to as *AtGH9C1*. This gene is one of three GH9 class C enzymes in *Arabidopsis* and its role is more intriguing because it is the most dissimilar of the three [8]. The other two, *AtGH9C2* and *AtGH9C3* have high sequence identity and constitute a duplicated gene pair positioned within duplicated DNA segments of the genome [16], [17], [18].

Here we analyze the spatial-temporal distribution of *AtGH9C1* message, determine the localization of AtGH9C1-GFP-fusion protein with and without the CBM49, and analyze a mutant with reduced *AtGH9C1* expression. Our results demonstrate that *AtGH9C1* is expressed primarily in root hair and endosperm cells and agree with results from public transcriptomes from these tissues. We established that the AtGH9C1 is a secreted protein and that the CBM confers localization specificity within the cell wall. A dendogram of all known GH9 class C genes in angiosperms demonstrates that AtGH9C1 and S1GH9C1 belong to different clades. Other genes that cluster with AtGH9C1 are also expressed preferentially in root hairs. The walls of hair cells, like the walls of the cellularized endosperm, are special, unlignified and short-lived. We suggest that AtGH9C1, and other members of the same clade, hydrolyze β-1, 4 glucan chains in a cell wall context that is different than the one recognized by S1GH9C1, and that AtGH9C1 plays a role in the development of the root hair and of the endosperm in *Arabidopsis*.

## Results

### AtGH9C1 is Expressed in Roots and Seeds of Arabidopsis

*AtGH9C1* is located on the upper arm of chromosome 1, (At1g48930) and encodes for a polypeptide 627 amino acids long, (69 kD, pI 9.18), that is moderately glycosylated. This gene is composed of 8 exons, of which the first one carries a predicted signal peptide [e.g. 19] and the last one encodes the entire CBM49 that is tethered to the catalytic core by a short polypeptide linker encoded in part by exon 7. The remaining exons encode the catalytic module.

**Figure 1:**
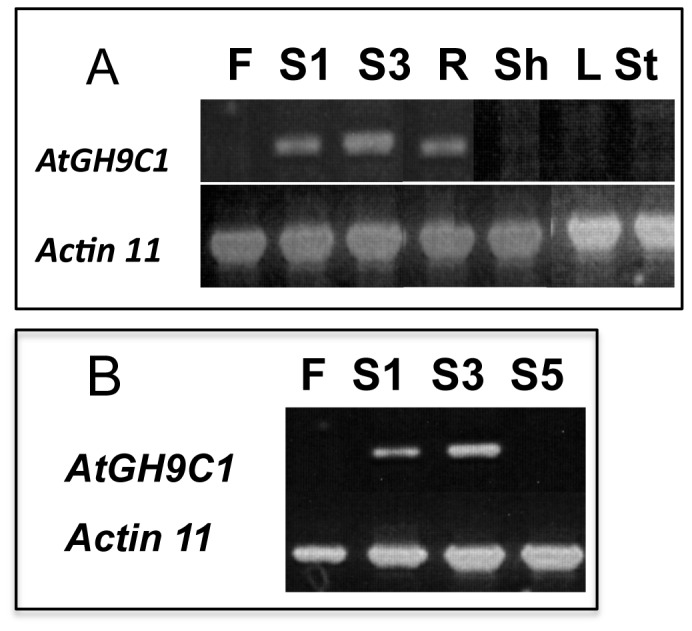
*AtGH9C1* expression in *Arabidopsis thaliana*. **(A)** Detection of *AtGH9C1* and *Actin 11* messages by RT-PCR, using as template total RNA isolated from flowers (F), young siliques (S1 and S3), roots (R), shoots (Sh), expanded leaves (L) and stem (St); (**B**) RT-PCR comparing flower (F) with three stages of silique development (S1, ∼2 mm long), S3, (<5mm long), S5, siliques reaching their maximal length (>1 cm long).

To gain a first insight into where *AtGH9C1* is expressed, RT-PCR was performed using a PCR primer pair based on the 6th and last *AtGH9C1* exons and RNA templates from major Arabidopsis plant organs. Results indicated that *AtGH9C1* is expressed abundantly in young siliques, and in roots (Fig. 1A). A band of low intensity was also observed in young shoots but bands were undetected in flowers, leaves and stem. Comparison of RT-PCR band intensity at 25 PCR cycles, (Fig.1B), also revealed that expression was absent in flowers, low in very young siliques (S1, ∼2 mm long), peaks at S3, (S3,<5 mm long), and was absent at S5 as siliques reach their maximal length (>1 cm long).

### Spatial-temporal Expression of AtGH9C1

**Figure 2:**
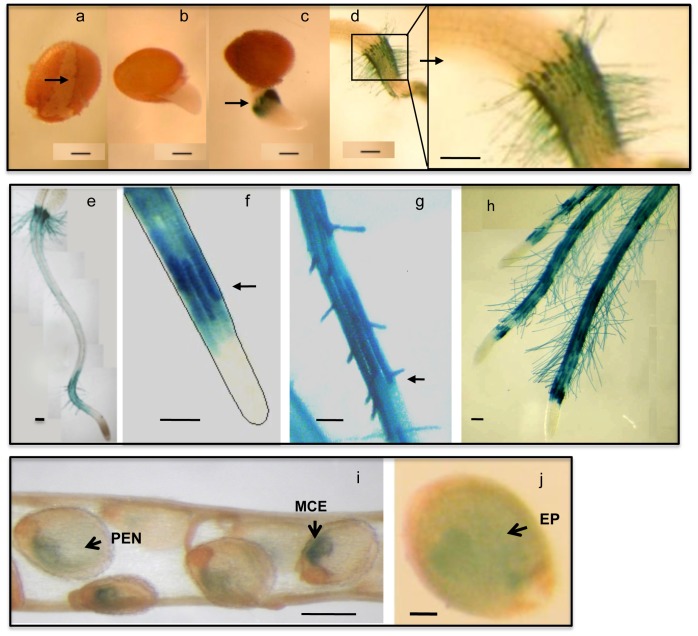
β-glucoronidase (GUS) activity driven by the *AtGH9C1* promoter in transgenic Arabidopsis. (**a**) Shahadara transgenic at 16–20 hours post light incubation (PLI), showing the seed coat ruptured (arrow). (**b**) 24–36 hours PLI showing seeds when the radicle has already emerged, and no signs of blue coloration. (**c**) 36–48 hours PLI showing strong blue coloration of GUS activity at emerging root hairs (arrow). (**d**) 72 hours PLI, showing the strong blue coloration at the collet zone (root-shoot junction) with a close up to the right. (**e)** 5 day old seedling showing strong GUS activity at the collet and at the differentiation zone of the main root. (**f)** close up of a main root tip showing GUS activity in trichoblast before the bulging and emergence of the root hair (black arrow). (**g**) emerging root hair proximal to the bottom cross wall of the trichoblast showing strong blue coloration**. (h)** Columbia transgenic seedlings growing in media supplemented with 1% sucrose with abundant and longer root hairs. (**i**) immature seed of a green silique positioned 1–1.5 cm down from the top flower bud, showing *AtGH9C1* expression in the micropylar endosperm (MCE), and toward the peripheral endosperm (PEN). (**j**) immature seed showing blue coloration in the embryo proper (EP) and the surrounding endosperm partially obscured by the encasing of the seed coat. Tissues were viewed in a light microscope at 40x. The bar lines in each photograph represent 200 µm.

**Figure 3:**
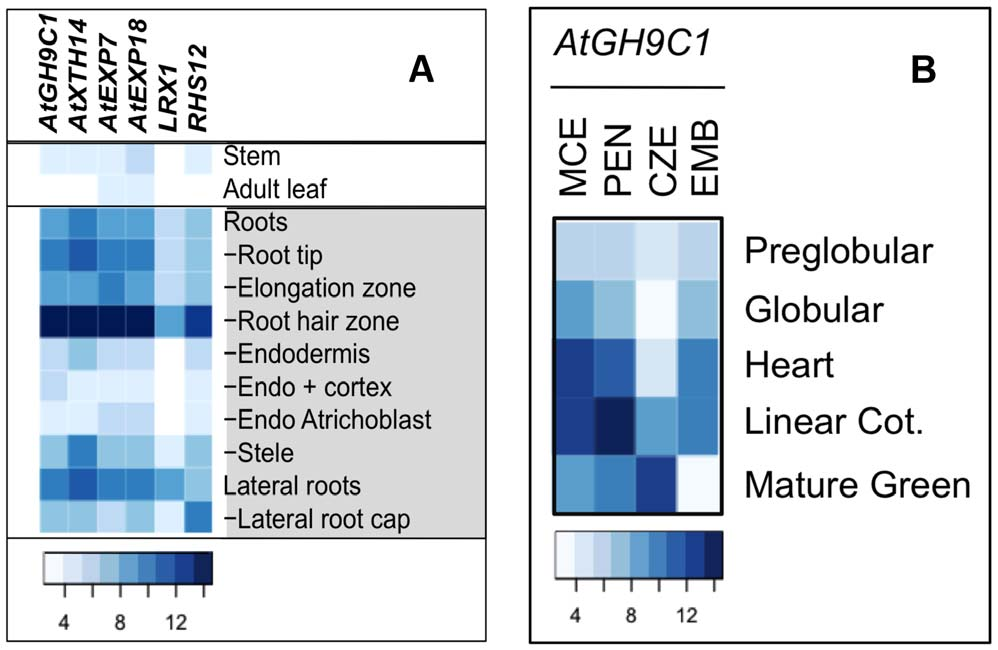
Anatomical distribution of *AtGH9C*1 expression in root and endosperm based on public transcriptome data. (A) Heat map showing level of expression (log base 2 transformed) of *AtGH9C1* and of five known root-hair specific genes, including, *AtXTH14*, (At4g28850), *AtExp7* (At1g12560), *AtExp18* (At1g62980), *LRX1* (At1g12040), and *SRH12* (At3g10710), for selected anatomical regions of *Arabidopsis* (data from Genevestigator [64]); **(B)** Heat map illustrating *AtGH9C1* expression (log base 2 transformed), in the micropylar (MCE), peripheral (PEN), chalazal (CZE) and the embryo proper, at five seed developmental stages, starting at the preglobular stage and ending at the mature green stage (data from http://estdb.biology.ucla.edu/genechip).

To examine expression of *AtGH9C1* with spatio-temporal resolution, its putative promoter (sequence of 1,600 bp preceding *AtGH9C1* coding region) was fused to the GUS reporter gene (*Escherichia coli* ß-glucuronidase) to generate *AtGH9C1::GUS* and used to transform *A. thaliana* Columbia, Lansberg and Shahadara ecotypes. Independent transgenic lines from each ecotype showed GUS activity in roots and young siliques, (Fig.2). Shahadara transgenic seeds were selected to monitor GUS staining at daily intervals after cold stratification, and transfer to continuous light. GUS activity was negative at 16–24 hours, when some seeds had a ruptured testa (seed coat) and some had an emerging radicle, (Fig. 2a and b). Between 30–72 hours, when elongation of the radicle continues, GUS activity was strong in all the epidermal cells of the collet, i.e., the root-shoot junction, (Fig. 2c and 3d). After 5 days, when cotyledons have emerged and the primary root is rapidly elongating, the strong GUS activity continued in the collet and extended to the differentiation zone of the main root (Fig. 2e). The blue staining was very strong only in the files of hair-forming cells (trichoblast), indicating low or no GUS activity in the atrichoblasts. Note that GUS staining was detected in the hair cell before the bulging and emergence of the root hair (Fig. 2f), and that the hair emerged closer to the bottom cross wall of the trichoblast (Fig. 2g), as described by others. Seedlings growing in media supplemented with 1% sucrose have longer root hairs and strong GUS staining in the hairs (Fig 2h). GUS activity was absent from the epidermal cells of the root cap and the meristematic region of the root tip.

GUS activity was also detected within the seeds of young siliques (silique # 2–3 from the top), in the compartments of the seed endosperm, particularly at the micropylar endosperm, (MCE), and the peripheral endosperm (PEN), (Fig. 2i), while older siliques displayed no stain. Note that even the embryo proper shows a weak blue coloration (Fig. 2j), revealed through the seed coat.

To substantiate our data, we searched Genevestigator, https://www.genevestigator.com for *AtGH9C1* gene expression in publically available transcriptomes. We confirmed that the *AtGH9C1* message was detected most abundantly at the root zone where the root hairs develop. We also confirmed the expression of AtGH9C1 in developing seeds noticing that *AtGH9C1* message is present in the three domains of the seed endosperm i.e. the MCE, the PEN and the chalazal (CZE).

### AtGH9C1 Expression is Comparable to Root Hair Marker Genes

Next, we expanded the Genevestigator search to compare *AtGH9C1* expression with cell-wall genes known to regulate root hair morphogenesis. A heat map was developed with this public data (Fig 3A) to illustrate that the abundance of *AtGH9C1* message, (column 1) in the root hair zone of the main and the lateral root, is comparable to that of root-hair marker genes, including endo-xyloglucan transglycosylases AtXTH14/XTR9, [4], [20], (column 2), expansins, AtEXP7 [21] (column 3), AtEXP18, (column 4), structural cell wall proteins, LRX1 [22], (column 5) and RHS12, a pectin esterase, (column 6). Similarly, and comparable to those root hair marker genes, *AtGH9C1* expression is lower in the stem, in adult leaves and in other root cell types including the endodermis, the cortex, atrichoblast and the stele.

### AtGH9C1 Expression in the Endosperm Follows an Anterior-posterior Polar Direction

We also searched the publically available transcriptomes of *Arabidopsis* endosperm before [23], and after cellularization (http://estdb.biology.ucla.edu/genechip. *AtGH9C1* expression was found in the proliferating syncytial endosperm, in the group of genes expressed preferentially in the endosperm, (“endosperm preferred”, EP, with a>2-fold differential expression in the endosperm sample compared with the silique sample) [23]. A heat map (Fig 3B) illustrates the progression of *AtGH9C1* expression level in the three domains of the endosperm and in the embryo proper through 5 stages of seed development (from preglobular to mature green embryo stage). *AtGH9C1* message is present in the MCE at low level at the globular developmental stage, and persists in the MCE at the heart developmental stage. *AtGH9C1* expression then extends into the PEN at the linear cotyledon stage, reaching here the highest level of message accumulation, and finally expands into the CZE at the green cotyledon stage. Thus, *AtGH9C1* expression seems to follow the sequential polar antero-posterior organization of endosperm cellularization and endosperm dissolution. There is also expression of *AtGH9C1* in the embryo proper at the heart and linear cotyledon stages [24]. Interestingly, and consistent with our analysis of GUS transgenic seeds during imbibition and germination, the transcriptomes data of seed germination indicated that *AtGH9C1* message level is very low and unchanged all through seed imbibition, rupture of the endosperm, breakage of the testa, and radicle emergence ( [25] Supplemental Dataset 1).

### AtGH9C1 Expression Responds Like a Root Hair Marker Gene

**Figure 4:**
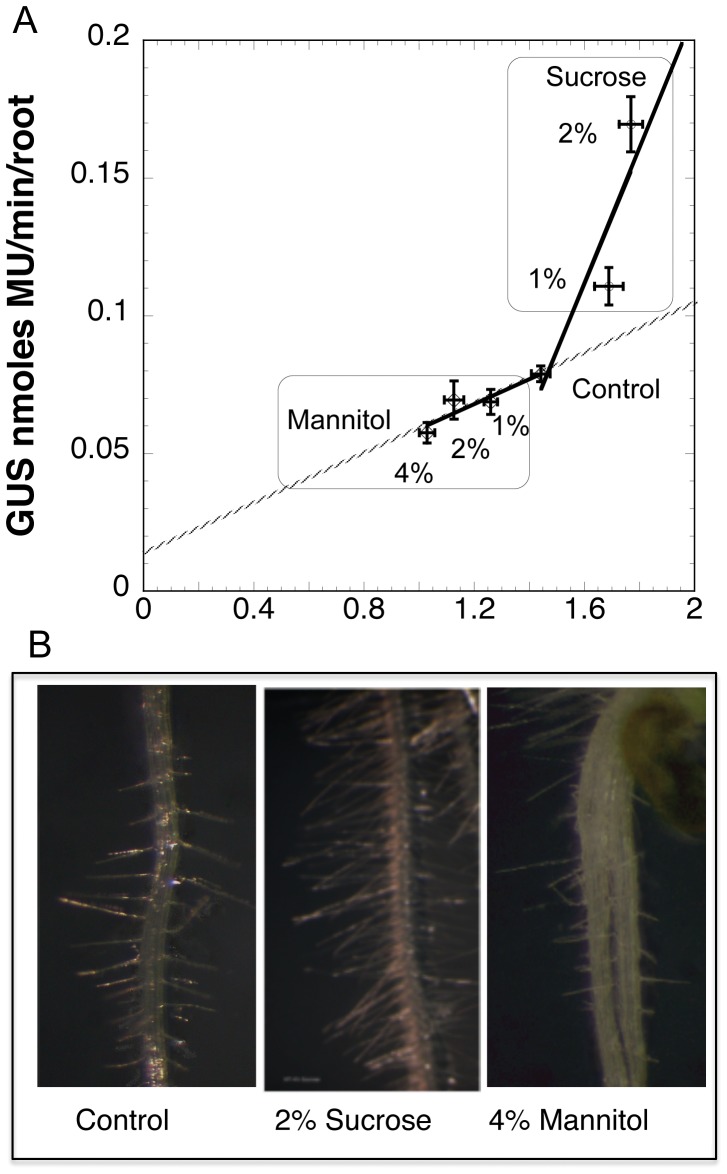
Effect of sucrose and mannitol on root length and *AtGH9C1::GUS* activity. (**A**) Transgenic seeds were stratified and grown on half-strength MS medium supplemented respectively with none, 1%, 2%, 4% mannitol, 1%, 2%, sucrose and maintained in continuous light at 22 C°. After 7 days, root length was measured using pictures of the plates and root GUS activity was determined on a known number of roots collected from the plates using MUG as substrate. Error bars show the standard error of MU production rate (y) and the standard error of mean root length (x). (**B**) Close up of wild type roots showing the effect of growing in control media or in media supplemented with 2% sucrose,or 4% mannitol.

If *AtGH9C1* expression is localized at the root hairs, GUS activity in the whole root of the transgenic lines should quantitatively correlate with root length, since longer roots will have more root hairs. Thus, seedlings of the transgenic line were exposed to treatments that generate either shorter roots (increasing concentration of mannitol) or longer roots (1% or 2% sucrose, [26]), over an equal growth period (7 days post illumination). The correlation between root length and GUS activity was confirmed in the mannitol treatment where both measurements were negatively impacted by the increasing osmotic stress imposed by mannitol (Fig 4A). Moreover, each of the measurements was reduced in about equal proportion, e.g. a linear regression between the two variables extrapolated to around zero. However, when transgenic seeds were germinated and maintained in control media supplemented with 1% or 2% sucrose GUS activity increased to a much greater degree than root length (Fig. 4A). In addition, seedlings grown in sucrose concentration had longer root hairs compared with seedlings in control or mannitol media (Fig. 4B). Thus, assuming that those treatments do not affect the number of root hair forming cells, the data suggests that AtGH9C1 contributes not only to the formation of the root hair but also to the lengthening of the hair.

**Figure 5:**
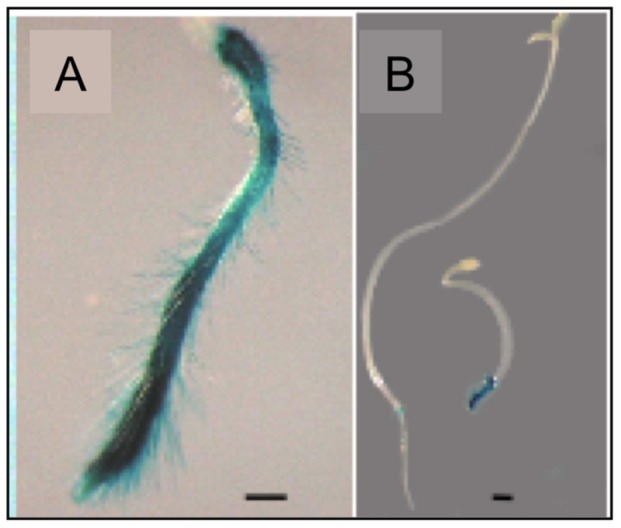
Effect of ethylene on *AtGH9C1* expression. **(A)** Transgenic *AtGH9C1::GUS* seedling (10 days old) growing in a media supplemented with 10 mM ACC and continuous light. (**B**) Etiolated, 5 days old, transgenic ethylene mutant *ers-2* carrying *AtGH9C1::GUS* (left) and transgenic wild type *AtGH9C1::GUS* (right), grown in media supplemented with 10 µM ACC and in the dark.

Another treatment known to affect root growth and root hair density is the plant hormone ethylene [27]. Thus, seeds of transgenic *AtGH9C1*::*GUS* (Columbia) were germinated in agar plates supplemented with 10 µM 1-aminocyclopropane-1-carboxylate (ACC), a substrate for ethylene synthesis, in both light and dark conditions. At 72 h post-germination in continuous light, seedling roots displayed an increase in root hair abundance as expected, and a strong blue coloration throughout the root (Fig. 5A). At 48 h post-germination in darkness, the transgenic seedling showed the triple response and a strong blue coloration in the root (5B-right). This contrasted with the very weak blue coloration observed in the root of the etiolated ethylene insensitive mutant (*ers-2*), also carrying the same *AtGH9C1*::*GUS* construct and germinated in darkness under the same conditions (Fig. 5B-left).

To further analyze the regulation of *AtGH9C1* expression by ethylene, transgenic *AtGH9C1::GUS* seeds were germinated in agar media supplemented with 10 µM ACC and kept in darkness for 3 days. After the treatment the whole seedling was used for GUS activity determination. Results showed a 5-fold increase in GUS activity in etiolated seedlings grown in the presence of ACC compared to those grown in control media (Table 1). The effect of 20 µM AVG, an inhibitor of ethylene synthesis, and the effect of both AVG and ACC, to test for inhibition reversal, was also analyzed in 3 day old transgenic seedlings, kept in continuous light. The activity from seedlings in AVG was around half the activity in ACC. The activity of seedlings exposed to both chemicals was less than the activity in presence of ACC alone. This indicated that simply adding ACC could not fully reverse the inhibition caused by AVG, as noted by others, [21]. Finally, the effect of ACC was dose dependent as shown by the analysis of GUS activity in 7 day old seedlings exposed to 0.1 µM, 1 µM or 10 µM ACC and kept in continuous light. Both shoot and root were analyzed separately and both showed a concomitant increase in GUS activity compared to seedlings grown on control plates (no ACC) and the activity was always greater in the root than in the shoot tissue (Table 1). Overall, these results indicate that *AtGH9C1* expression is regulated by ethylene, which increases root hair density (Fig 5A), [28].

**Table 1:** GUS activity (MU min^−1^ seedling^−1^) in transgenic Arabidopsis.

	Root	Shoot	Whole Seedling	
Treatment	light	light	light	dark
**3 days**				
Control				0.024±0.001
10 µM ACC				0.118±0.008
10 µM ACC			0.031±0.001	
20 µM AVG			0.016±0.001	
10 µM ACC+20 µM AVG			0.024±0.001	
**7 days**				
Control	0.037±0.001	0.008±0.000	0.045*	
0.1 µM ACC	0.179±0.030	0.016±0.000	0.195*	
1.0 µM ACC	0.201±0.001	0.077±0.001	0.278*	

* Activity in the whole seedling calculated by adding activity in the root and shoot.

Fluorometric determination of GUS activity using MUG as enzyme substrate and extracts from transgenic *AtGH9C1::GUS*. The activity values represent the slope of the regression of MU produced versus time of incubation for each treatment using the indicated tissue extract (±SE, N = 5). Treatments included light and dark germination and media supplemented with increasing concentrations of ACC (1-aminocyclopropane-1-carboxylate), or with AVG (Aminoethoxyvinylglycine) or with both ACC and AVG.

### AtGH9C1 Expression Follows the Pattern of Root Hair Marker Genes

The expression of *AtGH9C1* and of the same set of root-hair marker genes mentioned above was searched in public root transcriptomes that used treatments or genetic backgrounds that promote or reduce root hair development. A heat map (Figure S1), shows that different treatments that promote root hair growth over control treatment, such as iron [29], phosphorous [30], [31] and water deficiency, changed the expression of *AtGH9C1*and of the marker genes in similar ways. Particularly, all increased around two fold with phosphorous and water deficiency (red panels). In contrast, in mutant lines, that develop shorter root hairs, like *rsl4* [32] and *rhd2-2* [33], the expression of these genes decreased compared to wild type controls (green panels). Moreover, in a line that over expressed RSL4 (35S::RSL4) [32], and resulted in longer root hairs, *AtGH9C1* and the marker genes increased gene expression (GEO accession number GSE19530).

### Cell Wall Localization of AtGH9C1-GFP with and without CBM49 before and after Plasmolysis

**Figure 6:**
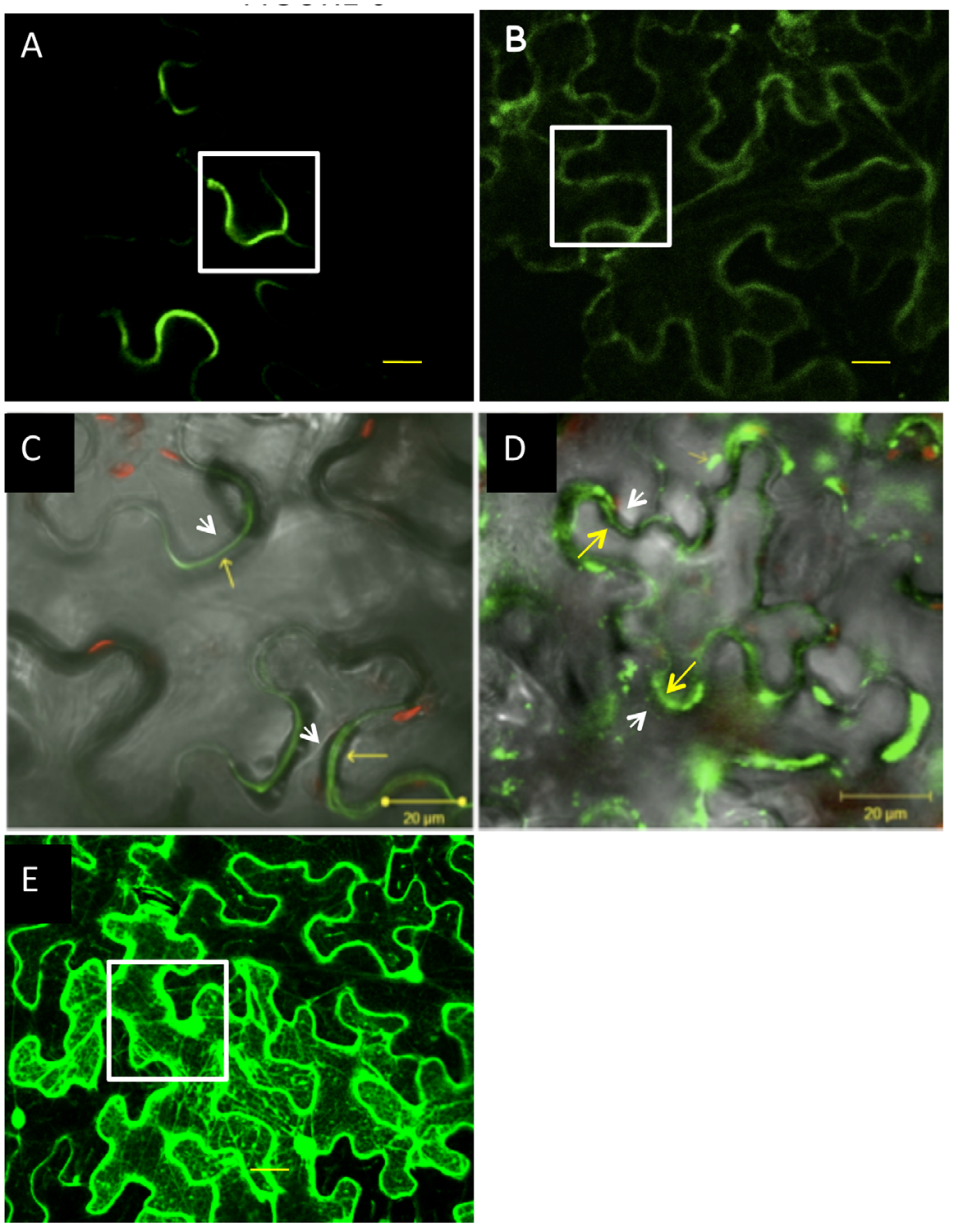
Transient expression driven by 2X35S and detection of C-terminal AtGH9C1-GFP, in epidermal leaves of *N. benthamiana*, before and after plasmolysis. (A, C) GFP fluorescence emitted by the AtGH9C1-GFP fusion construct with the CBM, (B, D) without the CBM, (E) without the AtGH9C1, i.e. a construct expressing the GFP alone as positive control. Images were viewed under a 63x water lens. Scale bars are 20 µm. (A, B) Green fluorescence before plasmolysis, (C, D) after plasmolysis and overlay with brightfield transmitted light image to show the integrity of the cell wall after the treatment (*gray illustrates the* bright field image). Inset white boxes in A and B highlight the difference in the fluorescence signal when the CBM is removed. Yellow arrows point to the fluorescence in the cell wall while the white arrows point to the position of plasma membrane after plasmolysis.

The deduced amino acid sequence of AtGH9C1 predicted the presence of a signal peptide at the N-terminus suggesting that the nascent polypeptide is imported into the endoplasmic reticulum and then secreted outside the cell. To elucidate experimentally the cellular localization of this protein and the role of the CBM in localization we generated fusions between the GFP and AtGH9C1 (with and without the CBM). The fusion protein was expressed using the 2X 35S promoter to guarantee a strong expression. Both, C-terminal AtGH9C1-GFP:±CBM, (Fig.6) and N-terminal (GFP-AtGH9C1:±CBM) (Figure S2) fusions were generated to test for possible interference from the GFP. Each construct was infiltrated into *N. benthamiana* leaves to induce transient expression and then the localization of the fluorescence protein (with and without the CBM) was analyzed before and after plasmolysis by confocal laser microscopy.

There was a clear difference in the distribution of fluorescence from AtGH9C1-GFP fusion with the CBM (Fig.6A) *vs.* without the CBM (Fig 6B). With the CBM, the fluorescence contours only some segments of the cell wall surface (Fig.6A), The discrete fluorescence segments were positioned in some neck and lobe regions of the epidermal cells and the intensity of the green fluorescence was high. In contrast without the CBM the fluorescence was diffused and dispersed throughout the epidermal cell wall (Fig. 6B).

When *N. benthamiana* leaves were infiltrated as above and subsequently incubated in 1M sucrose (instead of water), the localization of fluorescence from AtGH9C1-GFP with the CBM showed no signs of alteration despite the retraction of the plasma membrane (Fig. 6C). In contrast, the localization of fluorescence from AtGH9C1-GFP lacking the CBM became diffused accumulating in extracellular regions as blotches of random sizes (Fig. 6D). No fluorescence was observed in the cytosol in either case. This contrasted with the distribution of fluorescence from the 35S:GFP alone, (Fig. 6E) which appears inside and outlining each cell very strongly. Thus, secretion AtGH9C1 to the cell wall was confirmed for both AtGH9C1-GFP with and without the CBM and the localization was altered when lacking the CBM.

Although the fluorescence was weaker, the same pattern of localization was observed for the N-terminal fusion with and without the CBM, where the GFP was inserted between the signal peptide and the rest of AtGH9C1 (Figure S2). This infers that GFP does not interfere with the secretion of AtGH9C1 in either the C- or N-terminal fusions. Because of its more intense fluorescence, we used the C-terminal (AtGH9C1-GFP: ±CBM) fusions for further studies.

### Stable Over-expression Expression of AtGH9C1-GFP with and without CBM and Ectopic Localization AtGH9C1-GFP Protein in Arabidopsis Thaliana

To analyze the possible consequences of over expressing AtGH9C1-GFP with and without the CBM, we transformed wild type Arabidopsis with the C-terminal (*AtGH9C1*-GFP: ±CBM) fusion constructs. Although GFP was observed in the transformed lines, there were no obvious phenotypic difference among the over-expressor lines (T3 generation) and wild type. The studies included analysis of germination efficiency, and growth of root and hypocotyl, at 5 day-post germination (in light or continuous dark). Nevertheless, the images of GFP fluorescence of these lines illustrated again a different spatial distribution between AtGH9C1-GFP with and without the CBM (Figure S3). Most noticeable was the accumulation of GFP fluorescence in association with the stomata cavity only if the CBM is present.

### AtGH9C1-T-DNA Insertion Mutants: Phenotypic Characterization

**Figure 7:**
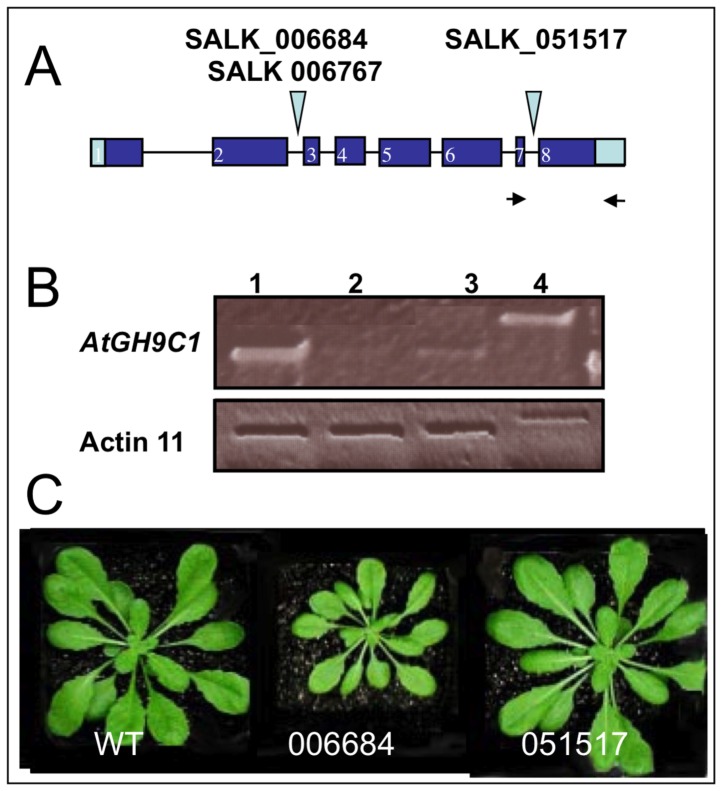
*AtGH9C1* gene structure and T- DNA insertional mutants. (**A**) Diagram of *AtGH9C1* gene with blue boxes representing exons, separated by introns, depicted by solid lines and position of the T-DNA in three mutant lines. Black arrowheads represent the position of primers used to detect AtGH9C1 message. **(B)** Detection of *AtGH9C1* message by RT-PCR using as template total RNA isolated form green siliques comparing wild type, (line 1), SALK 051517, (line 2), SALK 006684, (line 3), and wild type genomic DNA (line 4). RT-PCR of Actin 11 was used as an internal standard. Note that in both panels, genomic DNA yields a larger MW band because the primers used are separated by one intron. (**C**) Similar age wild type and homozygous, SALK 006684 and SALK 051517 growing in soil.

To gain further insight into *AtGH9C1* gene function, we obtained from the SALK collection three lines with a T-DNA insertion in *AtGH9C1* gene [34]. The SALK 006684 and SALK 006767 lines were indicated to have a T-DNA insertion at intron number two, while the line SALK 051517 in exon number eight (Fig.7A). Using gene-specific primers and a T-DNA left border primer, a homozygous mutant for each line was identified and the position of insertion confirmed by sequencing the DNA segment amplified from the T-DNA left border primer. Our sequence determined that the T-DNA insertion is intronic in SALK 006684 and SALK 006767, but indicated that in the SALK 051517 line the insertion was also intronic and toward the end of intron 6.

Expression of *AtGH9C1* in SALK 006684 and 051517 was further analyzed by RT-PCR using total RNA from green siliques and a primer pair based on exon seven (Fw) and on the 3′UTR (Rv). These primers amplify a 500 bp segment from the *AtGH9C1* cDNA transcript, (Fig. 7B lane 1, 2 and 3) and a 580 bp segment (Fig. 7B, lane 4) from genomic DNA. The results indicated that the 500 bp transcript band was clearly detected in wild type (lane 1), barely visible in SALK 006684 (lane 2), and detected in SALK 051517, though less intense than in wild type (lane 3). Thus, these lines are not true null mutants and both have a variable reduction on *AtGH9C1* gene expression compared to wild type, perhaps because the T-DNA insertion is removed when the introns are spliced out with different frequency depending on the insertion position.

**Figure 8:**
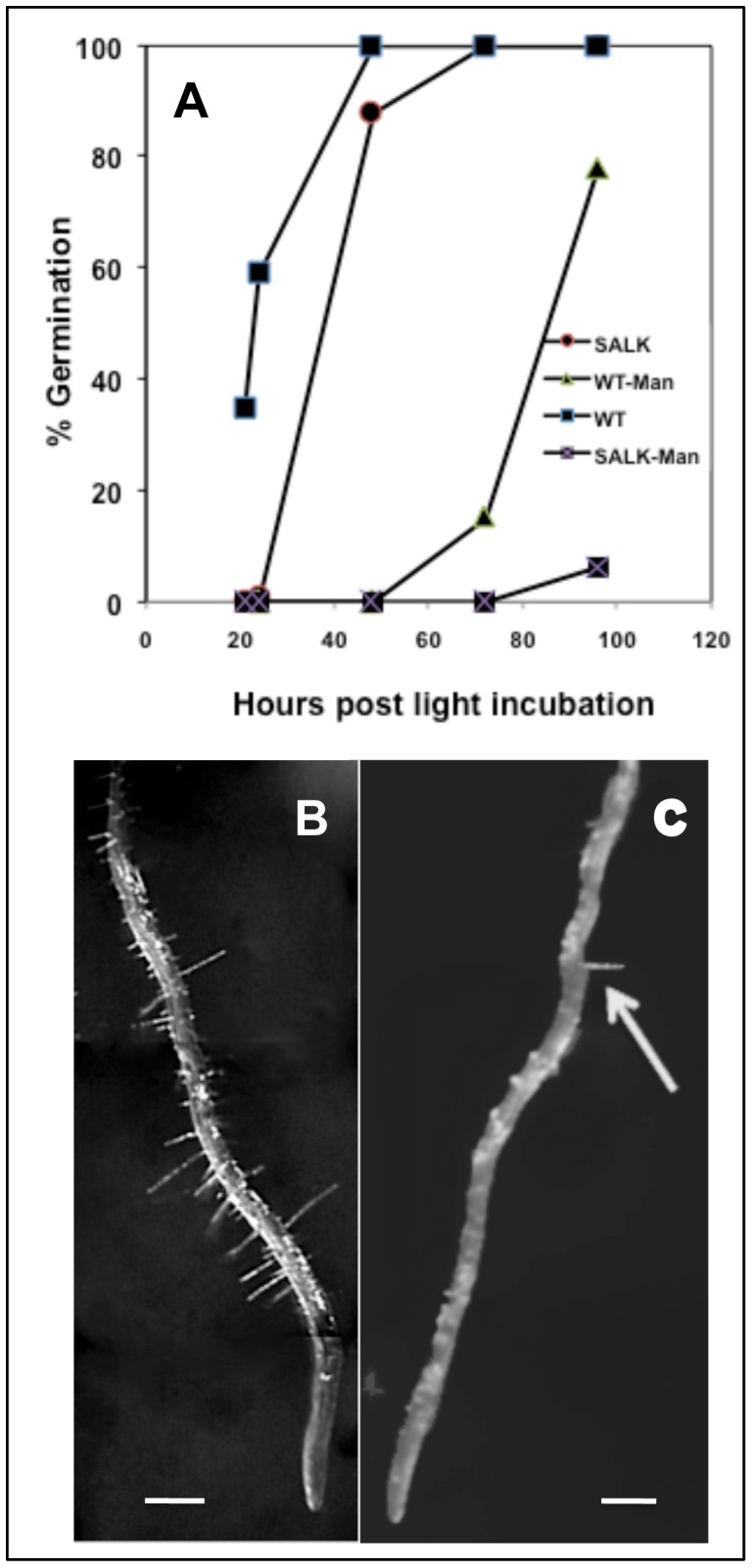
SALK 006684 phenotypic characterization. (**A**) Percent germination versus time of SALK 006684 (circles and crosses lines) and wild type Columbia (squares and triangle lines) using control media (circles and squares lines) or media supplemented with hyperosmotic 4% Mannitol (crosses and triangle lines). (**B**) Close up of a 7 day-old wild type root growing vertically on control medium. (**C**) Close up of a 7 day-old SALK 006684 root, growing vertically on control medium, with only one root hair visible.

**Figure 9:**
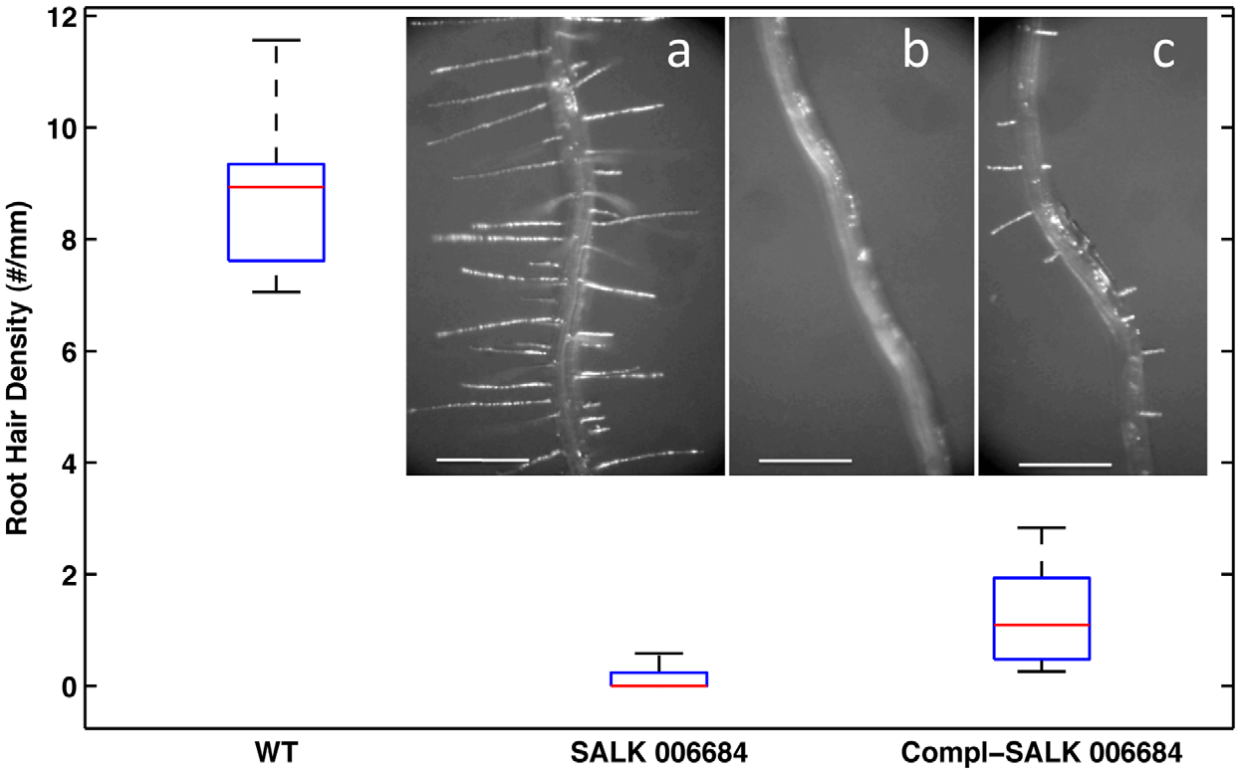
Box-whisker plot of root hair density for the *Arabidopsis* wild type Columbia, SALK 006684, and Complemented-Salk 006684. On the box for each Arabidopsis line, the red center line is the median root hair density, the top and bottom edges of the blue box are the 25^th^ and 75^th^ percentiles of the distribution, and the black whiskers extend to the most extreme data points. Insets, close up of a root segment above the zone of root hair differentiation to illustrate the root hair density and length of (a) wild type, (b) SALK 006684, and (c) Compl-SALK 006684. Pictures were taken at 40X magnification. Lines represent 500 µm.

In general, growing in soil, both mutant lines germinated, produced normal plants and abundant seeds, yet SALK 006684 plants consistently displayed a reduction in rosette size compared to wild type and SALK 051517 line (Fig.7C). Therefore, growth of SALK 006684 was further characterized on 1/2MS agar plates always comparing with wild type. Seeds of SALK 006684 showed a delay in germination of 12h–24h, and the delay became even more evident (36h–48) when the agar media was supplemented with hyperosmotic 4% Mannitol, (Fig. 8A). SALK 006684 and wild type showed no apparent differences in root hair abundance or root hair length at the collet or at the main root, 72 h post germination. However, once the root began to grow down the agar plate, the main root of the mutant showed a noticeably lower root hair abundance compared to wild type (Fig 8B-C). The average root hair abundance for wild type, few millimeters above the zone of differentiation was ∼ 9 hairs/mm while for the mutants was ∼ 0.2 hairs/mm (Fig. 9). This suggests that the root hair phenotype becomes visible when a low activity threshold for AtGH9C1 is reached.

Other studies comparing wild type with SALK 006684 included a comparison of root length growth in control media, over a 7-day period post-light incubation, (Figure S4A). The time course demonstrated consistently that the wild type roots were longer than those of the mutant, and that the absolute differences between them remain about the same over the 7-day period of growth. Therefore, the difference in root length observed was a reflection of the delay in germination and not due to a reduced rate of root growth. A comparison in a media supplemented with sucrose to stimulate root hair growth (Figure S4B) demonstrated clearly the root hair abundance difference between the two lines. Note that the mature SALK006684 plants (Figure S4C, left) have small stature compared to wild type (Figure S4C, right), and that some siliques from the main stem were shorter and defective (Figure S4D). Results of equivalent experiments performed with SALK 051517 had similar tendencies but differences were smaller.

Finally, a feature common to both mutant lines was that only 1% of the seeds germinated compared to 50% of wild type seeds after four years of storage in a dry cabinet. These results suggested that both mutants’ seeds lost viability faster than those of the wild type and that *AtGH9C1* expression in the endosperm may contribute to seed longevity during storage.

### Mutant Complementation Test

Subsequently, we complemented the mutants with the wild type gene to examine whether the reduction of *AtGH9C1* expression in the mutants contributed to the delay in seed germination and to the reduction in root hair abundance and length. Complementation was accomplished by transformation of the homozygous SALK 006684 and SALK 051517 lines with a construct carrying the full-length AtGH9C1-GFP driven by the double 35S promoter (referred to as Compl-SALK 006684 and Compl-SALK 051517). The selected complemented lines used for phenotypic characterization were hygromycin resistant, GFP positive, and either homozygous or heterozygous dominant because gene expression under the 2X 35S promoter confers a dominant character [35]. Three phenotypic characters, % germination and root hair abundance and length were analyzed. In each study, the comparison included the wild type, the mutant and the complemented mutant.

Two independent trials were conducted to measure percent (%) germination of seeds at 24 h post-light incubation, (Table 2). In both trials, the % germination of SALK 006684 improved significantly with complementation, (Tukey HSD test, *p* = 0.05−0.01, respectively), but only in trial two did reach the % germination level of the wild type. For SALK 051517, the % germination was not different compared to the wild type, and although the % germination of Compl-SALK 051517 was slightly higher than the wild type, the difference was not statistically significant (Table 2). We concluded that the complemented lines were only partially rescued by the fusion construct with the GFP and that the fusion enzyme may have reduced enzyme activity. Nevertheless, they also supported the hypothesis that *AtGH9C1* plays a role preparing the seed for germination during the period of high expression in the endosperm.

**Table 2:** SALK lines Complementation Test based on % seed germination.

SEED TYPE	Trial #1(%)	#Seeds	Trial #2(%)	#Seeds
WT	87.3^a^	244	89.3^a^	214
SALK 006684	21.8^b^	211	44.1^b^	211
Compl-SALK 006684	39.6^c^	114	85.0^a^	100
SALK 051517	86.8^a^	114	93.1^a^	101
Compl-SALK 051517	95.6^a^	114	95.0^a^	100

Percent seed germination of wild type, SALK Lines and SALK lines expressing AtGH9C1-GFP (with the CBM) driven by the 2X35S promoter. Seeds were distributed in control MS-agar plates and cold stratified. The percentage of seeds showing radical protrusion were counted after 24hs of transferring the plates to a 20°C light incubator. Letter superscripts denote groups that are significantly different from each other based on the Tukey HSD test for multiple comparison of proportions, *p* = 0.01, except for group c in Trial #1 the difference between SALK 006684 and Compl-SALK 006684 has *p* = 0.05.

Analysis of root hair abundance and length, were conducted in 7 day-old seedlings growing on control agar plates (Fig. 9). While we could find “patches” with hairs on the roots Compl-SALK 006684 (Fig. 9b Inset), it was not easy to verify the increase by visual observation alone. Therefore, we systematically counted the root hairs on pictures of consecutive mm of root segments (see details in *Methods*) to quantify an average density. Density was quite variable between roots and within a root, with a range (min to max) of 7–11.6 hairs mm^−1^ for WT and 0.3–2.8 hairs mm^−1^ for Compl-SALK 006684 (Fig. 9). Nevertheless, the complemented mutant had a significantly greater average root hair density than the mutant (t-test, *n* = 14, *p* = 0.013). As mentioned above, complementation did not totally rescue the WT phenotype, in terms of root hair abundance and length (Fig. 9c inset). The average (±SD) root hair length for wild type was 0.48±0.22 mm while for the Compl-SALK 006684 was 0.21±0.1 mm.

### Phylogeny of CBM49 from Higher Plants and Lower Photosynthetic Species

**Figure 10:**
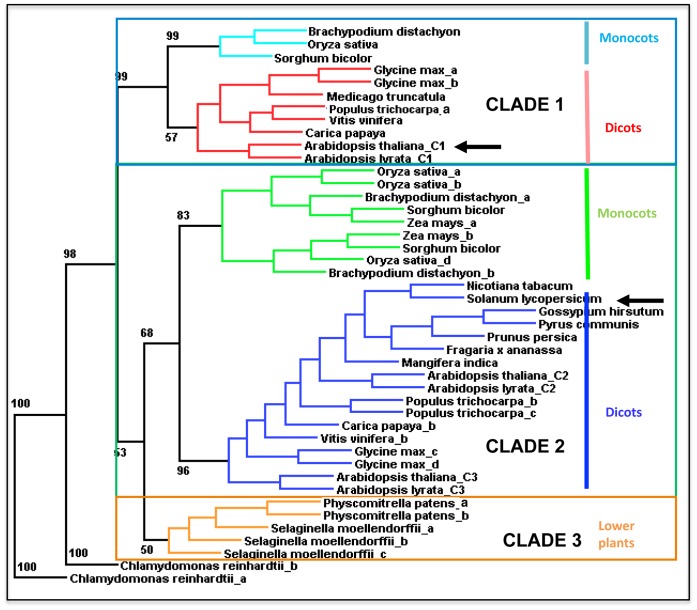
Phylogeny and clade organization of proteins endo-β 1,4 glucanases with a C-terminal extension. A rooted consensus tree was constructed from the deduced amino acid sequences of angiosperms and lower plant endo-β 1,4 glucanases with a C-terminal extension. Protein sequences were aligned using CLUSTAL, which was subsequently input into *Phylip* (see further details in Materials and Methods) to perform maximum likelihood phylogeny reconstruction. Arrows mark AtGH9C1 in CLADE 1 and S1GH9C1, in CLADE 2. The different proteins in the tree are identified by species followed by a letter suffix to distinguish multiple members from the same species. For *Arabidopsis* the suffix distinguishes the three members of GH9 class C. The branches of the tree are color coded to denote the monocot and dicot members of CLADE 1 and CLADE 2. The specific protein sequence accessions numbers and locus identification for each gene (http://www.ncbi.nlm.nih.gov/) are presented in Table S1.

All sequenced genomes of higher plants and early photosynthetic species such as *Physcomitrella*, *Selaginella* and the unicellular algae *Chlamydomonas* contain GH9 genes coding for endo-β-1,4-glucanase possessing a C-terminal extension. The specific protein sequence accessions numbers and locus identification for each gene are presented in Table S1. The phylogenetic tree, using *Chlamydomonas* as a root, shows that the proteins from lower photosynthetic species form a separate clade (Clade 3) that is distinct from the two other clades (Clade 1 and 2) formed by genes from higher plants (Fig. 10). Each clade from higher plants includes members of the monocot and dicot plants.

S1GH9C1, the tomato GH9 with a CBM49 at the C-terminal extension that was shown to bind crystalline cellulose [15] is placed in Clade 2. The *Arabidopsis* AtGH9C1 and its paralogue in *A. lyrata* also possessing a CBM49 at the C-terminal extension are both placed in Clade 1. Instead, AtGH9C2 and AtGH9C3, and corresponding paralogues of *A. lyrata* are all placed in Clade 2, of which several are expressed in fruit [15], [36], [37].

It’s apparent then, that GH9 enzymes with C-terminal extensions from Clade 1 are distinct from those in Clade 2 and this distinction (based on percent amino acid identity) may represent differences in protein structure and possibly functional differences. The difference in amino acid composition between members of Clade 1 and Clade 2 is illustrated in the amino acid alignment of dicot GH9 with C-terminal extensions (Figure S5). The C-terminal extension of members of Clade1 are enriched in glutamine (∼9%) while, members of Clade 2 are enriched in serine (∼20%).

### Expression Profile of Other GH9C-type Enzymes from Clade 1

To determine if other enzymes from Clade 1 have same functions as AtGH9C1 we searched for their expression profile in public microarray databases. The *Medicago truncatula* Gene Expression Atlas indicates that *MtrGH9C1* of Clade 1 (http://mtgea.noble.org/v2/probeset.php?id=Mtr.39531.1.S1_at&submit=Go) is exclusively expressed in root hair cells. Also, the expression of the two soybean members of Clade1 (Glyma11g10760 and Glyma12g03050) had been analyzed along segments of the main root by quantitative RT-PCR using primers that amplify both genes. The highest expression of these genes occurs in the root segment 2–7 mm behind the root meristem where cell elongation occurs and root hair initiation begins. At each side of this segment the expression is lower and is undetected in leaves (Dr. Tucker, unpublished results). Unfortunately, probes for these two soybean genes are not included in the GeneChip microarray of microdissected soybean seeds (http://seedgenenetwork.net/). Efforts to have global transcriptome profiling of every tissue in Poplar (http://www.popgenie.org) and Sorghum [38] are in progress. From the data already available, we found that the corresponding GH9 members of Clade 1 from these two plants (POPTR_0007s07790.1 and Sb06g032760) are both preferentially expressed in the root over shoots with some expression in young leaves and nodes in Poplar.

## Discussion

*AtGH9C1* is one of the three endo (β-1, 4) glucanase (GH9) genes of *A. thaliana* possessing both a catalytic module and a carbohydrate-binding module, family 49 (CBM49), at the C-terminus. Here we demonstrated that *AtGH9C1* is expressed primarily in root hairs and endosperm cells. Both cell types have specialized unlignified, short-lived cells walls that are eventually weakened and fully dismantled.

### A Role for ATGHC1 in Root Hair Development

The tubular outgrowth of root hairs from trichoblasts involves a polarized secretion of enzymes and addition of new cell wall polysaccharides at the growing apex [39], [40]. These molecules bring about the concurrent localized loosening and reinforcement of the root hair cell wall [41]. Several proteins contribute to cell wall loosening in *Arabidopsis* root hairs, including expansins [21] and xyloglucan endo transglycosylase-hydrolases (XTHs) [20], [42], [43], [44], [45]. Our data demonstrate that *AtGH9C1* constitutes another molecular player in *Arabidopsis* root hair growth and is expressed before the initiation of the bulge at the trichoblasts, and contributes to the elongation of the hair cell (tip growth) in coordination with other cell wall enzymes.

The specific expression of *AtGH9C1* in the root hair was clearly demonstrated by our transgenic lines carrying the promoter *AtGH9C1::GUS* construct and by published root microarray experiments that analyzed the differentiation zone where root hairs develop. GUS activity was observed in the trichoblast committed to the development of hairs, both before hair initiation and after the hair formed. This activity was increased by treatments that increased root hair density (ACC), increase root length and root hair length (sucrose) and decreased by treatments that reduced root length (mannitol).

A role of *AtGH9C1* expression in root hair development was further supported by published microarray experiments that included the concomitant response of *AtGH9C1* expression to treatments that, a) increased root hair length (P and Fe deficiency); or b) mutants with short or defective root hairs (*rhd2, rsl4*) [33], [46]. Specifically, in the *rhd2-1* mutant, the expression of *AtGH9C1* is reduced 58% compared to wild type, which is comparable to the reduction observed for the root-hair specific expansins [33]. Finally, a role of *AtGH9C1* expression in root hair development was also supported by the root hair phenotype of the homozygous SALK 006684 mutant, which became visible when a low activity threshold for AtGH9C1 was reached.

*AtGH9C1* responds to ethylene signaling and its transcriptional regulation includes several transcription factors that control root hair growth and elongation such as RSL4, a basic helix-loop-helix (bHLH) [32]. By searching the transcriptome of RSL4, we found that the expression of *AtGH9C1* followed a pattern of gene expression consistent with the regulation of root hair formation by RSL4. More recently, the transcriptome data of *upbeat* [46] become available and showed that *AtGH9C1* expression was 15 times reduced compared to wild type in the root elongation zone. UPBEAT1 is a transcription factor that regulates the balance between the zones of cellular proliferation and cell elongation [46]. Since our *AtGH9C1::GUS* transgenic lines showed strong reporter activity in the root-hair cell before bulge formation, it suggest that *AtGH9C1* expression is de-repressed by UPBEAT1 once the cell has reached the transition zone between cell elongation and root hair differentiation.

### A Role for *AtGHC1* in Endosperm Development

In addition to expression in root hair, RT-PCR demonstrated *AtGH9C1* expression in young siliques while our GUS transgenic established a localization of the expression to the seed endosperm. But the connection of this expression with the development of the seed endosperm was revealed by reference to the public transcriptome series of Arabidopsis seed development (before and after endosperm cellularization). The endosperm is an specialized tissue that provides the essential nutrients for the development of the embryo and the carbon needed for the synthesis of wall polysaccharides of the new embryo cells [47]. Some of this carbon is derived from catabolic reactions occurring in the cell walls of the mature endosperm, which in many seeds functions as a carbohydrate-storing compartment. However, in *Arabidopsis*, the cell walls of the endosperm are transient and thin. They contain limited amounts of cellulose, high amounts of hemicellulosic polysaccharides and they are totally dismantled to give room to the growing embryo [48]. The breakdown of the endosperm compartments occurs after cellularization, following a polar anterior-posterior sequence. It starts in the MCE (late heart stage) even before the PEN and the CZE are cellularised. Once the PEN cellularizes, it persists only for a few days, degenerating when the embryo progresses from the linear cotyledon to the green cotyledon stage [49]. Similarly, the CZE, which is the last region to cellularize, degenerates partially as the embryo matures to the green stage [50], [51]. *AtGH9C1* expression is found in all three endosperm compartments and seems to follow the progression of cellularization and dissolution in each compartment. The characterization of SALK 006684 demonstrated that reducing AtGH9C1 activity contributes to delayed seed germination and reduced seed viability. Thus, AtGH9C1 could be involved in the process of gradually breaking down the endosperm cell walls as the embryo grows. Moreover, since *AtGH9C1* begins to be expressed prior to, or at, the onset of MCE cellularization, it is likely that AtGH9C1 activity is present even before the wall is fully formed. Thus we speculate that AtGH9C1may contribute to maintain a thin and easily degradable wall of the endosperm cells.

### Different Localization of AtGH9C1-GFP with and without the CBM

The deduced amino acid sequence of AtGH9C1 predicted the presence of a signal peptide at the N-terminus. Results after plasmolysis of *N. benthamiana* leaves, transiently expressing AtGH9C1-GFP with and without the CBM, confirmed that AtGH9C1 protein is secreted and demonstrated that the CBM immobilized the protein to specific regions of the cell wall. Transgenic *Arabidopsis* constitutively expressing AtGH9C1-GFP also displayed a different localization of the fusion protein when the CBM was absent.

### Two Clades of CBM 49

Phylogenetic analysis, based on amino acid sequence similarity to AtGH9C1, showed that GH9 proteins with CBM49 modules exist in a wide range of photosynthetic organisms from lower species such as algae, ferns and mosses to higher plants. This suggests an important and conserved role for the CBM49 of GH9 enzymes. The tree shows two different Clades (Clade 1 and 2) for the angiosperms while the lower photosynthetic species form a distinct Clade from higher plants (Clade 3). The two Clades of higher plants indicate two subtypes of GH9 proteins with CBM49 due to some differences in amino acid composition. The biological significance of these subtypes is unknown but they could represent differences in specificity or possibly functional differences, among them. Their broad occurrence in angiosperm suggests that they play a role in plant development perhaps because they confer the enzyme’s ability to recognize distinct forms of a substrate common to all cell walls. The CBM49 characterized in the tomato cellulase (SlGH9C1) by Urbanowicz et al. [15], binds crystalline cellulose and belongs to Clade2, while AtGH9C1 belongs to Clade1. Crystalline cellulose is found in all plant cell walls. Apart from this ordered physical form, cellulose is also found in more disordered, amorphous forms, at the surface of small cellulose microfibrils and when forming complex structures with other wall polysaccharides [13]. Cellulases containing cellulose-binding modules with binding specificities for different cellulose forms exsit in microbes and fungi, [10], [52], [53]. Thus, if it is assumed that the CBM49 in both clades bind cellulose, we speculate that the distinction between clades might reflect their ability to recognize different cellulose forms. *AtGH9C1* is expressed in root hairs and endosperm cells. Other genes that cluster with *AtGH9C1* are also expressed preferentially in root hair cells. The walls of hair cells, like the walls of the cellularized endosperm, are special, unlignified and short-lived. The cellulose of these walls is probably like the cellulose found in the primary wall that is expanding and consists of smaller microfibrils that could bear between 33–80% of amorphous cellulose [13], [14].

The binding of the CBM to cellulose depends on the prevalence and location of the planar side chains of aromatic residues at the protein-ligand interface. This allows hydrophobic stacking with the planar crystal cellulose [10], [15]. In addition in the *C. fimi* Cel9B CBM 4–1, some glutamine and asparagine have been shown to contribute to the affinity of towards soluble sugars and soluble cellulose derivatives [54]. The CBM49 of members of Clade1 and Clade2, have the same number and position for conserved tryptophans and tyrosines, but members of Clade1 are enriched in glutamine (∼9%).

So the question remains as to which cell-wall substrate AtGH9C1 hydrolyzes, to which ligand the CBM binds and what is the distinctive aspect of this substrate where AtGH9C1 is active. When transiently expressed, AtGH9C1 is localized in some lobe-neck regions of the pavement cells where a restricted outgrowth is coordinated and compensatory to the expansion of the adjacent cell [55], [56]. So our results indicate that the substrate of AtGH9C1 is either localized in selected regions of the wall, or it is localized in all regions but is being modified only in specific areas that the CBM49 of AtGH9C1 recognizes. If these localized regions are expanding, this substrate is either amorphous cellulose or a glucan polymer that AtGH9C1 can modify to facilitate cell-wall weakening. Alternatively, as proposed by Shpigel et al. [57], the binding of the CBM49 to the glucan chains may inhibit crystallization of microfibrils and thus weakening the wall to facilitate expansion. However, we cannot rule out that AtGH9C1 could bind to a different type of polysaccharide such as callose or hemicelluloses.

### Conclusions

AtGH9C1 is a secreted endo β-1,4 glucanase that has, in addition to the canonical cellulase catalytic site, a carbohydrate binding domain (CBM49). The CBM49 associated with this gene targets the protein to some regions of the wall and when the CBM is missing, the protein is diffusely distributed throughout the wall. Whatever the substrate may be, AtGH9C1 plays a role in root hair and endosperm development in *Arabidopsis*. We suggest that AtGH9C1 recognizes a form of cellulose or glucan polymer that is prevalent in the wall of root hair, in the endosperm and in the growing regions of the cell wall and that is different than the one recognized by S1GH9C1.

## Materials and Methods

### Plant Material and Growth Conditions

Seeds of *Arabidopsis* ecotypes Columbia, Lansberg or Shahadara (as indicated) were germinated in half-strength MS [58] basal salts (Sigma, St. Louis, MO), 0.5 g/L 2-[N-Morpholino] ethanesulfonic acid (Sigma), pH 5.7, supplemented with 1X Gamborg’s vitamins and solidified with 1% (w/v) plant tissue culture agar (Type E, Sigma). Seeds were surface-sterilized, cold treated for 4 days at 4°C and then placed vertically in a growth chamber (Conviron) at constant 20°C under 16-h light/8-h-dark regime. For germination in soil, seeds were dispersed over wet soil (Metro-Mix 300, Scotts Company), cold treated for 4 days at 4°C and grown to maturity in a growth chamber at 20°C under 16-h light/8-h-dark cycle. *Nicotiana benthamiana* seeds were planted in the same manner but cold stratification was not performed.

### Cloning Vectors

AtGH9C1 cDNA was obtained from Riken Bioresource Center in a RAFL-14-97-C17 plasmid (pda 12555). The pSTblue-1 vector was obtained from Novagen and the plant overexpression vectors, pMDC45 (stock no. CD3-739) and pMDC83 (stock no. CD3-742), were obtained from ABRC, through The Arabidopsis Information Resource (TAIR; Arabidopsis.org). Plasmid pBI 101.3 was a gift from Dr. P. Bottino.

### GUS Promoter-reporter

A chimeric construct was generated by fusing the putative AtGH9C1 promoter in frame with the GUS gene. The AtGH9C1 promoter was derived from Arabidopsis genomic DNA amplified with a forward primer that contains a BamHI restriction site at the 5' end and a reverse primer with a SmaI restriction site at the 5′end. The PCR product contained 1,600-bp of the putative promoter plus the 5′ UTR of the AtGH9C1 gene. This fragment was cloned first into PCRII vector (Invitrogen) and then cut at the SmaI and BamHI restriction sites. The fragment was purified and fused upstream of the GUS gene in the pBI101.3 plant transformation vector opened at its BamHI/SmaI site. A plasmid containing the promoter fragment was selected based on restriction size digestion and confirmed by DNA sequence. The construct also contains the NPTII gene that confers kanamycin resistance and was delivered to Columbia, Lansberg and Shahadara wild-type plants via Agrobacterium transformation by the floral dip method [59]. Seeds derived from transformed plants were selected in germination media containing kanamycin. The AtGH9C1/GUS construct was introduced also in the ethylene insensitive *ers*-2-2 mutant by crossing it with the AtGH9C1/GUS transgenic in Columbia background. Seeds from F2 generation were screen in half-strength MS agar plates containing 10 uM ACC in the dark and seedlings that showed the tall hypocotyl phenotype were selected and assayed for GUS activity in the presence of 10 uM ACC. The primers used to clone the promoter were Fw-5′AAGGATCCAGGATTCAAATGGGTGGACAAAAGA3’; Rv-5′ AACCCGGGTTCTCTATGTCAATCTAAGTTCTAA3’.

### Histochemical GUS Analysis

Transgenic seedlings and siliques were collected in 90% acetone, incubated on ice for a few minutes, rinsed with 50 mM NaPO_4_ pH 7.2, 0.5 mM K_3_Fe(CN)_6_ and 0.5 mM K_4_Fe (CN)_6_, and then placed in staining solution 2 mM X-Glu; (5-bromo-4-chloro-3-indolyl-β-D-glucopyranoside from Sigma), dissolved in rinse solution. They were vacuum infiltrated for 10 min and incubated at 37°C for 2 h or overnight. Seedlings were examined using a microscope and photographed with a Digital Nikon 990 Camera.

### GUS Spectrophotometric Assay

For quantification of GUS activity, a fluorogenic substrate, 1 mM MUG (4-methyl-umbelliferyl-β-D-glucuronide), was used in the assay according to the method of Jefferson et al. [60]. Root and shoot tissues were collected and extracted to analyze GUS activity from transgenic AtGH9C1-GUS seedlings growing vertically in 1/2MS agar plates for 7 days. For the analysis of the effect of mannitol, sucrose or ethylene, the half-strength MS medium was supplemented with either 1%, 2%, 4% mannitol, 1%, 2% sucrose, 0.1 µM, 1 µM, and 10 µM of 1 aminocyclo-propane-1 carboxylic acid (ACC, Sigma), 20 µM AVG (aminoethoxyvinylglycine, Sigma) made from a stock (1,000X) prepared in water. Tissues were ground in reaction buffer 2∶1 (v/w) and centrifuged for 5 min at maximal speed in a microfuge to clear the supernatant. An aliquot of crude extract was mixed with an equal volume of reaction buffer containing 2 mM MUG and incubated at 37°C. Aliquots were taken at 15 min intervals and the reaction was stopped with 0.2M Na_2_CO_3_. Fluorescence was determined using a one-channel fluorometer (Turner Design Picofluor) with an excitation range of 365-395 nm, emission wavelength greater than 430 nm and calibrated with known concentrations of 4-methylumbelliferone (MU). The fitted linear slope (± standard error) of MU produced vs incubation time was used as relative measure of GUS content for comparison with control treatments.

### RNA Isolation and RT-PCR Analysis

For tissue specificity studies, total RNA was isolated from 200 mg of different plant tissues (buds, green siliques, rosette leaves, stems and roots) as described in the RNA Plant Mini Kit (Ambion). A two-step RT-PCR was performed using the Retroscript kit (Ambion, Austin, Texas). Total root RNA was pretreated with DNAse prior to reverse transcription (Ambion DNA-free kit). First strand cDNA was prepared with oligo dT primers and used as template for PCR reactions. RT-PCR was normalized using Actin-11 primers. Primers for AtGH9C1 were: RV- 5′GACAAAAAAGCATTCTCTACCAAGG3’ based on the 3′UTR; Fw-5′AAACCTTATGAAACTACAAAA-CCAG3’ based on the 7^th^ exon. Primers for Actin-11 were, Fw-5′-ATGGCAGATGGTGAAGACATTCAG-3′; Rv-5′GAAGCACTTCCTGTGGACTATTGA-3′. Each PCR reaction was run for 25 or 30 cycles with a denaturation step for 45 sec at 94°C, and an extension for 70 seconds at 72°C, Annealing was for 30 seconds at optimal temperature for each primer pair: AtGH9C1 48°C; Actin-11, 60°C.

### N-terminal and C-terminal GFP Fusion of AtGH9C1 with and without CBM

For N-terminal GFP fusions the signal peptide was fused upstream of the GFP followed downstream by the rest of the *AtGH9C1*cDNA (with and without the CBM). For the C-terminal GFP fusions, the *AtGH9C1*cDNA (with and without the CBM) was fused upstream of the GFP after removing the stop codon and the 3′ UTR from the *AtGH9C1*cDNA. To prepare the C-terminal-GFP fusion, the pMDC83 vector was used. DNA segments of *AtGH9C1* cDNA, with and without the CBM were amplified by PCR and cloned upstream of the *GFP*. The Fw primer was based on the 5′UTR of *AtGH9C1*. The Rv primer including the CBM was based on *AtGH9C1* last exon, excluding the stop codon (UAG). The Rv primer for the segment without the CBM was based on the last 20 bases of exon six. Forward and reverse primers were designed with terminal restriction sites, BamHI and KpnI, respectively, for subsequent cloning into pMDC83.

To prepare the N-terminal-GFP fusion, the pMDC45 vector was used. A stitching PCR approach was required to fuse the predicted signal peptide (SP), (MRKFGGSLFGVSLLLSVLLAAATAAÂEYYN) of AtGH9C1 (SignalP 3.0) upstream to the GFP gene and then fuse, downstream of the GFP, the rest of the *AtGH9C1*cDNA (with and without the CBM). This would allow for the secretion of the fusion protein to the cell wall. The ^∧^ symbol represent the site for the endopeptidase.

The GFP gene was amplified from pMDC45 using Fw and Rv primers. The Rv primer to stitch the *AtGH9C1* signal peptide to the N-terminus of *GFP*, and the Fw primer to stitch the C-terminus of the GFP to the rest of AtGH9C1cDNA were both 60 bp ULTRAMERS™. The Fw and Rv primers to stitch all pieces together to be cloned into pMDC45 included, a KpnI and a PacI restriction sites, respectively. The Rv primer designed to amplify *AtGH9C1* without the CBM included the sequence preceding the C-terminal extension, a stop codon and a Pac I restriction site. All primers were designed using Integrated DNA technologies (IDT) PrimerQuestSM and they are listed in Table S2 indicating the pair that was used for each specific reaction.

### PCR Amplification and PCR Stitching Protocol

Plasmid minipreps were diluted 1∶10 times before using as template DNA. Each standard PCR reaction mixture was composed of deionized H_2_O, 1/10th volume Thermopol buffer (NEB), 250 µM dNTP mixture, 240 pM forward primer, 240 pM reverse primer, 2.5 mM MgCl_2_, 2.5U Taq Polymerase (NEB) & approximately 50ng DNA. The reactions were run for 20 cycles with denaturation at 94°C for 30 seconds, annealing at Tm appropriate for primers for 30 seconds and elongation at 72°C for 3 minutes. Following amplification, gel electrophoresis and extraction from the gel, the three fragments: SP-GFP, GFP and GFP-AtGH9C1, were used as templates in a modified PCR reaction that included 50ng of each fragment, 24pM of the terminal primers and 2.5U Taq Polymerase as above. The PCR stitching fragment was confirmed on a 0.6% gel, isolated and subcloned into a pSTblue-1 vector (Novagen) blue according to the manufacturer’s instruction. The clones containing the stitching fragments (SP-GFP and GFP-AtGH9C1) were sequenced to confirm that were fused in frame to yield the expected amino acid sequence. Sequencing was performed on a Biosystems DNA Sequencer model 3730 (UMBI DNA sequencing facility) using a T7 promoter primer (1 pM/µl). See the list of primers used in Table S2.

### Ligation into pMDC45 or pMDC83

The pSTblue-1 and plant transformation vectors were restricted with the appropriate restriction enzymes: (KpnI+PacI) for pMDC45 and (BamHI+KpnI) for pMDC83. The reactions were run on an agarose gel and the needed bands were excised and purified. The appropriate fragments were ligated over night at 16°C in a 10 µl final volume for each reaction. The reaction mixture included 1/10th volume vector and 3/10th volume insert, T4 DNA ligase (2000 cohesive end units; NEB) and 1x ligase buffer (NEB). Each ligation reaction was used to transform competent high efficiency DH10B E.coli cells (NEB). Plasmids from individual colonies were isolated and restricted with KpnI+EcoRI for N-terminal-GFP fusion clones or with BamHI and KpnI, for C-terminal-GFP fusion clones.

### Generating Agrobacterium Competent Cells

N-terminal-GFP and C-terminal-GFP fusion plasmids were each introduced into competent GV3101 *Agrobacterium tumafaciens*, plated on LB agar (1.5% w/v Difco bacto agar) containing kanamycin (50 µg/ml), gentamycin (25 µg/ml) and rifampicin (50 µg/ml) and incubated at 30°C. Individual colonies were screened by colony PCR, in which after touching and boiling for 5 minutes, the supernatant (2 µl) was used as template for PCR reaction with the appropriate primers.

### Plant Transient and Stable Transformation with AtGH9C1-GFP Fusion Constructs

*Nicotiana benthamiana* plants (4–5 week old) were used to examine transient expression of the GFP constructs in leaf epidermal cells [61]. The GV3101 strains carrying the appropriate clones were grown on Luria broth (LB) overnight at 30°C, with the appropriate antibiotics. Three ml of each culture were centrifuged at 6000 rpm for 15 minutes and the pelleted cells resuspended in infiltration medium (10 mM CaCl2, 10 mM MgCl2, pH 5.7) to an O.D. of 0.8. Then, acetosyringone (3′, 5′-Dimethoxy-4'-hydroxyacetophenone) in DMSO (Phytotechnology Laboratories) was added to the suspension at 70 µM final concentration and left at room temperature for 3 hours without shaking. The abaxial side of 1.5–2 inch leaves was infiltrated using a 1 ml syringe (BD integra) supporting the adaxial side of the leaf with the paper card. The infiltration medium bled into the leaf forming a ∼0.25 inch diameter blotch. The plant was placed under natural sunlight for 48 hours with watering and infiltrated areas were observed with a Zeiss LSM-510 confocal microscope.

For stable *A. thaliana* transformation, GV3101 strains carrying the GFP fusion clones were grown in culture, and used as recommended for floral dip transformation [59]. Seeds were screened on ½ MS media agar plates with hygromycin (40 µg/ml). At least 5 independent transformant lines were collected for each and F2 generation seeds were used for further studies. Transformation was confirmed in seedlings by surveying GFP florescence using an EPI fluorescence micrscope.

### Root Length Measurements

Seedlings were grown in control half-strength MS media using large petri dishes (150 mm × 15 mm), kept vertically for up to 10–12 days. The dish base was divided into two half so that the two Arabidopsis lines to be compared were growing in the same dish. Photographs of the plates that included a ruler were taken to determine mean root length (±SE, N = 30) using ImageJ software.

### Root Hair Density and Length Measurements

Root hair density was determined on the main root using photographs of the main roots at 40X magnification. Efforts were made to maintain the roots growing over the surface of the agar, to keep as much as possible the roots separated from each other and maintain constant humidity while taking the pictures to avoid collapsing the root hairs. The root hairs longer than 0.05 mm were counted in 1 mm consecutive intervals starting at 3–5 mm from the tip to exclude the zone of elongation and the zone of root hair formation containing root hairs at different developmental stages. The root length surveyed was 3–4 mm for wild type and up to 6–8 mm for the mutant and the complemented line. The analysis included around 8 roots per line. Differences in mean root hair density between the mutant and the complemented line were tested for significance using ANOVA. Although the root hair length and density changes along older segments of root where fewer root hairs are visible, the newer segments, if the root is growing outside the agar media, have turgid and abundant root hairs that can be easily detected with a microscope.

### Confocal Laser Scanning Microscopy (Zeiss LSM-510)

LSM-510 image browser software was used under expert mode. The argon laser was used at 50% power. The Plan-Neofluar 10x/0.3 objective lens was used to visualize the image at 10x magnification under transmitted light. After the sample was focused the transmitted light was turned off and the blue excitation light with a GFP filter was turned on. Once the desired image was observed, image scanning (‘SCAN’ icon) was performed with the argon laser (488 nm). The ‘FITC dual with Rhodamine’ single track configuration (‘CONFIG’ icon) was used to scan the GFP signal (BP505-550 emission filter) as well as the red chlorophyll autofluorescence (LP 560 emission filter). The transmitted light image was scanned simultaneously through a different channel. A ‘Single Scan’ with line averaging of 8 was used to obtain the final image. A C-Apochromat 63x/1.2 W corr water objective lens (Zeiss) was used to image samples at 63x magnification.

### Phylogeny of GH9 with C– terminal Extensions

The amino acid sequence of AtGH9C1was used as queries to BLAST search 14 fully sequenced plant genomes plus genes from partially sequenced plants described in the Carbohydrate Activating Enzyme database (Phytozome 4.0 and CAZy protein database). A percent identity multiple sequence alignment was generated using CLUSTALW [62] at http://www.ebi.ac.uk/Tools/clustalw2/index.html. Seqboot was used for a 1000 data set bootstrap resampling of the CLUSTALW data. The Consense file was used to draw a rectangular phylogram in Dendroscope [63]. Protpars was used to obtain the most likely unrooted phylogenies using the parsimony method. Consense was used to obtain a rooted consensus tree from the Protpars multiple phylogeny output (1000 datasets) and input into Phylip to construct a rooted consensus tree (http://www.ebi.ac.uk/Tools/clustalw2/index.html). Chlamydomonas was used as a root because it is considered the most distant photosynthetic organism to land plants.

## Supporting Information

Figure S1 **Gene Expression in response to treatments that affect root hair.** Heat map (based on public root transcriptomes) showing the up-fold changes (red) in gene expression of *AtGH9C1* and of other root hair genes (columns) in the root hair zone [65], [66], after treatments (rows) that promote root hair development like iron and phosphorous deficiency (- Iron, -P) and salt (+Salt). Genes expression fold decrease (green) in the mutants, *rhd2* and *rsl4,* where the mutation cause a reduction in root hair presence. The expression of *AtGH9C1* and of the other root hair marker genes is restored in the RSL4 line, which complements *rsl4* and cause an increase in root hair length. Data for *rhd2* mutant taken from Jones *et* al., 2006 [33] and for *rsl4* and 35:RSL4 taken from Yi, *et* al., 2010 [32].
(TIF)

Figure S2 **Transient expression driven by 2X35S and detection of N-terminal AtGH9C1-GFP, with and without the CBM, in epidermal leaves of** ***N. benthamiana*** **.** Epidermal leaves of *N. benthamiana*, expressing GFP-AtGH9C1, (A) with the CBM; (B) without the CBM; (C) without the AtGH9C1 as positive control. Panel 1, chlorophyll autofluoresence; Panel 2, GFP fluorescence; Panel 3, leaf surface captured by transmitted light; Panel 4, overlay of the leaf surface with GFP and red fluorescence. Epidermal cells were observed using a confocal laser scanning microscope (Zeiss LSM-510) with 488 nm excitation and an emission range 505-550 nm. GFP localization shows accumulation of protein AtGH9C1 with the CBM in only segments of the cell wall surface, while localization of AtGH9C1 without the CBM is seen throughout the epidermal cell wall surface (B). Images A and B were taken with a C-Apochromat 63x/1.2 W corr (63x magnification. Image in C was taken with a Plan-Neofluar 10x/0.3 (10x magnification).
(TIF)

Figure S3 **Transgenic Arabidopsis expressing C-terminal AtGH9C1-GFP fusion construct with or without the CBM.** (A) fluorescence from AtGH9C1-GFP with the CBM on root hairs at different developmental stages. (B) fluorescence from AtGH9C1-GFP without the CBM illustrating fluorescence at the base and throughout the root hairs; (C, E and F) etiolated cotyledons showing fluorescence from AtGH9C1-GFP with the CBM in stomata; (D) etiolated cotyledons showing fluorescence from AtGH9C1-GFP without the CBM in the vascular traces and through-out all cells. (F) GFP overlay on the surface of etiolated cotyledons shown in E (40X); (F inset), Optical cross section through an open stomate showing GFP signal within the stomate cavity.
(TIF)

Figure S4 **Wild type versus SALK 006684 Characterization**. (**A**) Root length of SALK 006684 (yellow triangles) and wild type Columbia (red square) were measured daily during a week of growth in control agar plates, starting a day after transfer to 20°C; inset illustrate the difference between both lines, 5 days after transfer to 20°C. (**B**) Wild type and SALK 006684 growing in media supplemented with sucrose. Note the presence of abundant root hairs in wild type compared to seedlings of SALK 006684. (**C**) Plant stature of wild type (left) and SALK 006684 (right) growing in soil with regular watering for 6 weeks. (**D**) Inflorescence and siliques from the main stem of wild type (top) and SALK 006684 (bottom)
(TIF)

Figure S5 **Amino acid alignment of dicots GH9 with a C-terminal extension (Blosum 62) illustrating the conserved amino acids in black, the unique amino acids of CLADE 1 in red and the unique amino acids of CLADE 2 in green.**
(TIF)

Table S1 **Accession Numbers of proteins used in the GH9C Type dendrogram.**
(XLSX)

Table S2 **Primers used for stitching fragments to generate N-terminal and C-terminal GFP fusion constructs.**
(XLSX)
