# Supplementary figures and images for: A Tale of Two Tissues: AtGH9C1 Is an Endo-β-1,4-Glucanase Involved in Root Hair and Endosperm Development in Arabidopsis
Source: PLoS One. 2012 Nov 16;7(11):e49363. doi: 10.1371/journal.pone.0049363 (PMC3500288; doi:10.1371/journal.pone.0049363)

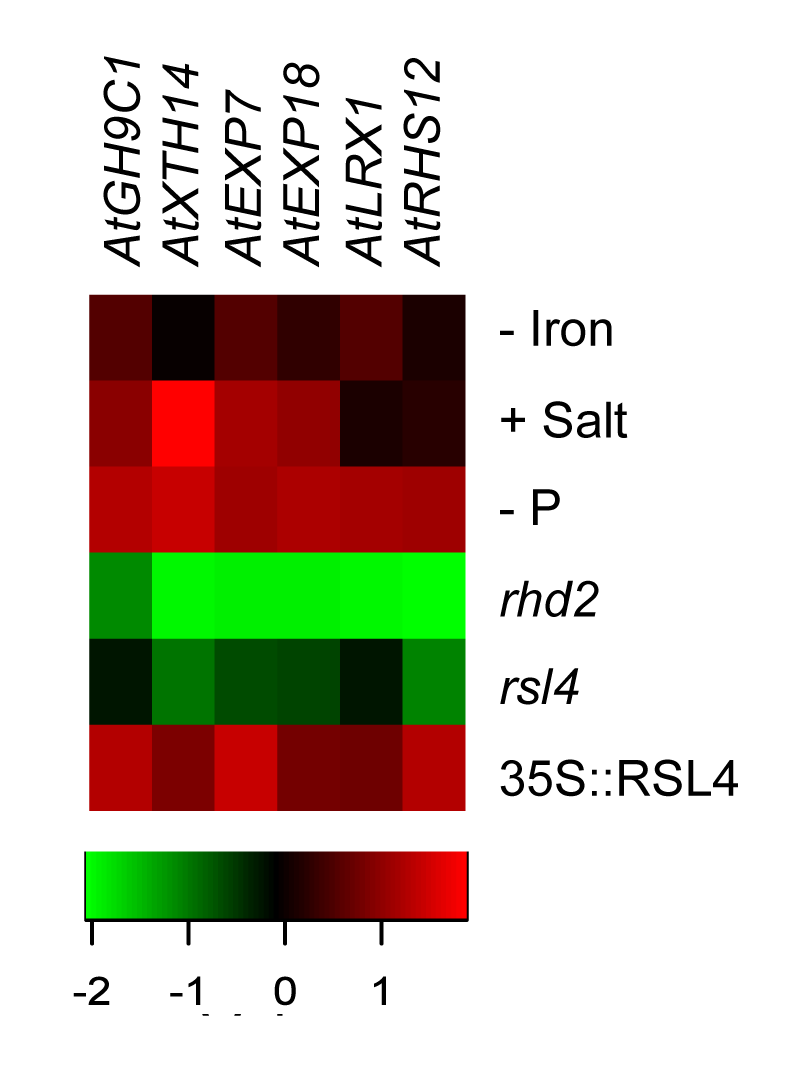

Supplement: Figure S1 — Gene Expression in response to treatments that affect root hair. Heat map (based on public root transcriptomes) showing the up-fold changes (red) in gene expression of AtGH9C1 and of other root hair genes (columns) in the root hair zone [65], [66], after treatments (rows) that promote root hair development like iron and phosphorous deficiency (- Iron, -P) and salt (+Salt). Genes expression fold decrease (green) in the mutants, rhd2 and rsl4, where the mutation cause a reduction in root hair presence. The expression of AtGH9C1 and of the other root hair marker genes is restored in the RSL4 line, which complements rsl4 and cause an increase in root hair length. Data for rhd2 mutant taken from Jones et al., 2006 [33] and for rsl4 and 35:RSL4 taken from Yi, et al., 2010 [32]. (TIF) [file pone.0049363.s001.tif]

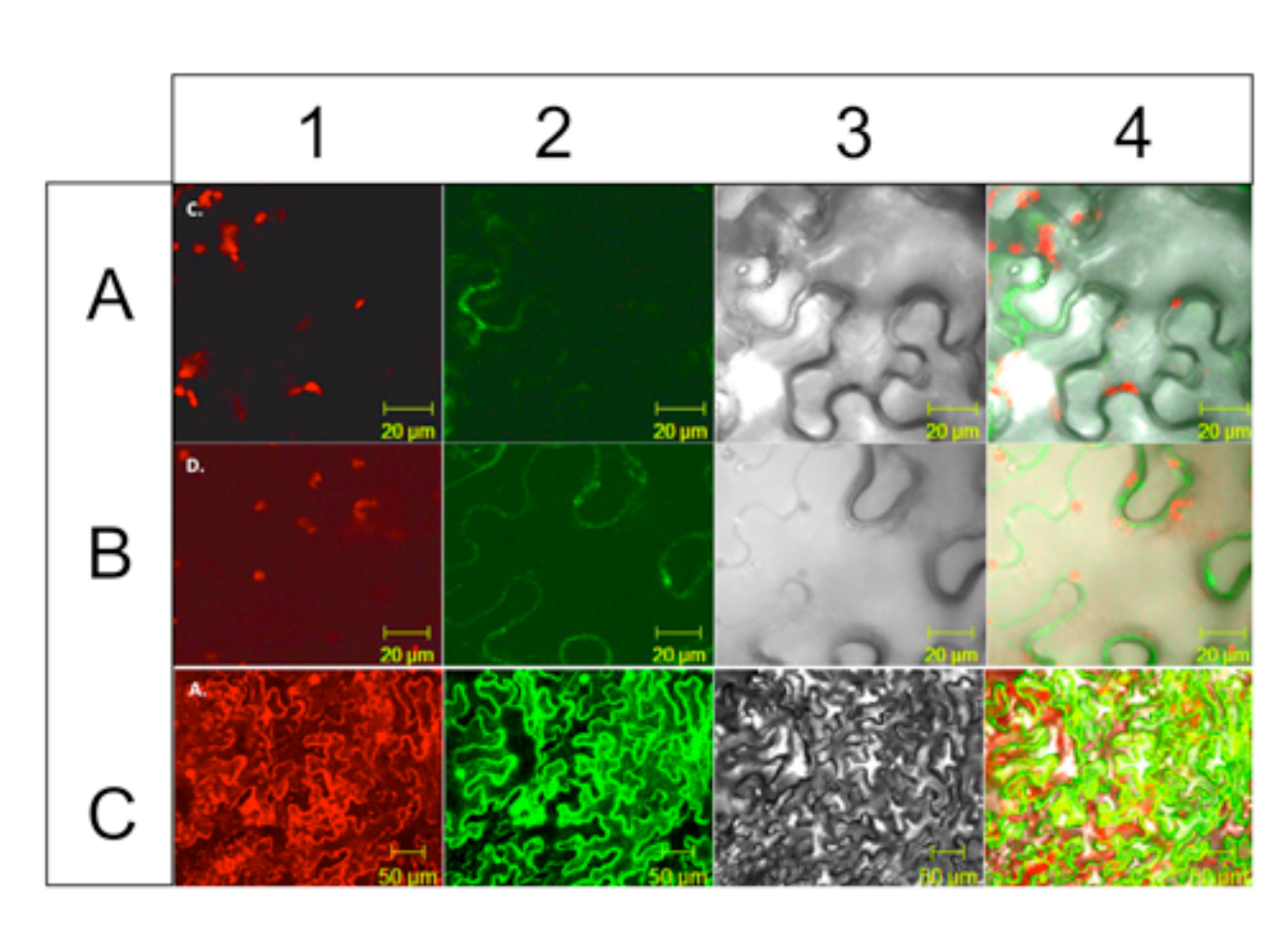

Supplement: Figure S2 — Transient expression driven by 2X35S and detection of N-terminal AtGH9C1-GFP, with and without the CBM, in epidermal leaves of N. benthamiana . Epidermal leaves of N. benthamiana, expressing GFP-AtGH9C1, (A) with the CBM; (B) without the CBM; (C) without the AtGH9C1 as positive control. Panel 1, chlorophyll autofluoresence; Panel 2, GFP fluorescence; Panel 3, leaf surface captured by transmitted light; Panel 4, overlay of the leaf surface with GFP and red fluorescence. Epidermal cells were observed using a confocal laser scanning microscope (Zeiss LSM-510) with 488 nm excitation and an emission range 505-550 nm. GFP localization shows accumulation of protein AtGH9C1 with the CBM in only segments of the cell wall surface, while localization of AtGH9C1 without the CBM is seen throughout the epidermal cell wall surface (B). Images A and B were taken with a C-Apochromat 63x/1.2 W corr (63x magnification. Image in C was taken with a Plan-Neofluar 10x/0.3 (10x magnification). (TIF) [file pone.0049363.s002.tif]

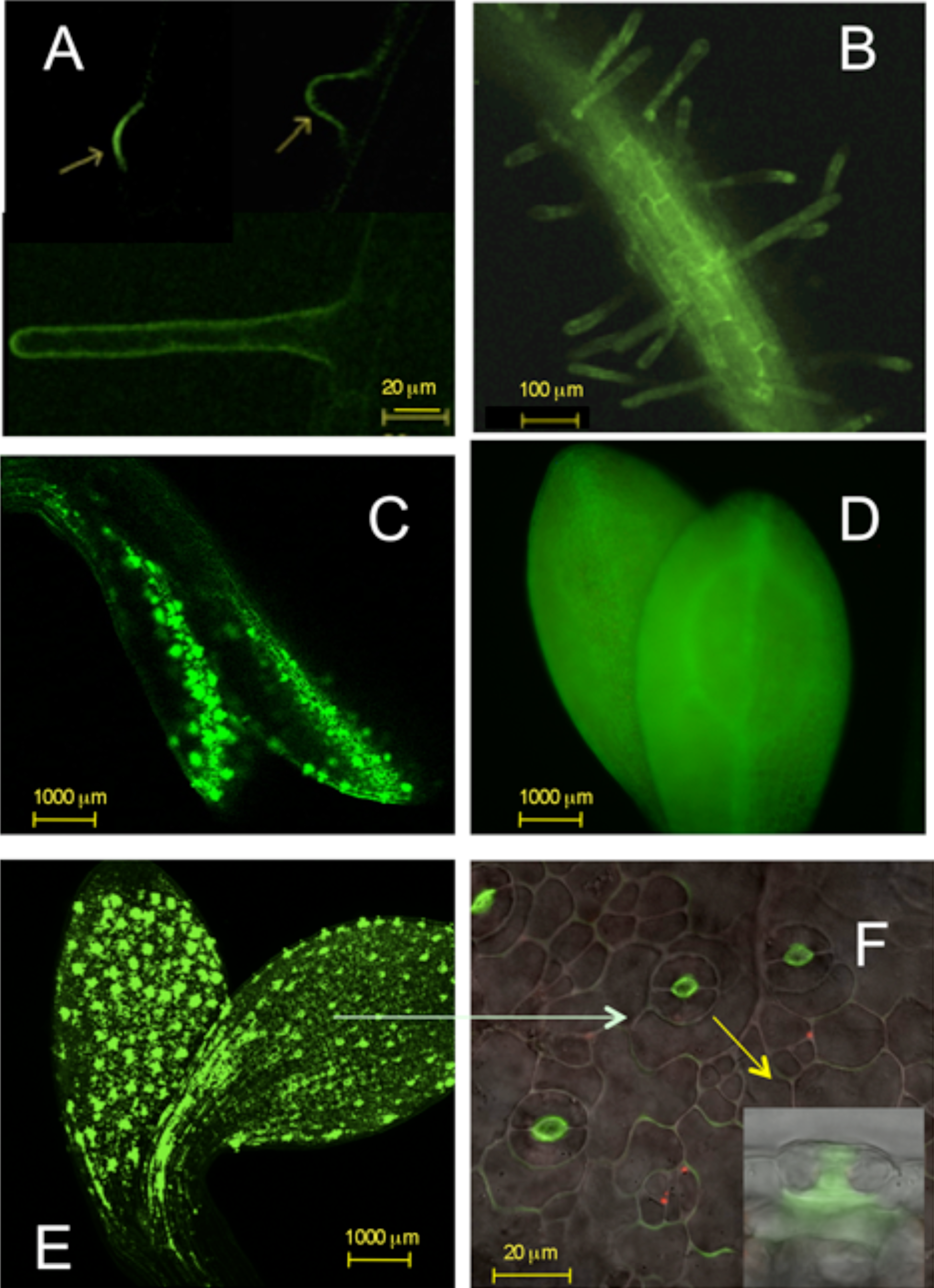

Supplement: Figure S3 — Transgenic Arabidopsis expressing C-terminal AtGH9C1-GFP fusion construct with or without the CBM. (A) fluorescence from AtGH9C1-GFP with the CBM on root hairs at different developmental stages. (B) fluorescence from AtGH9C1-GFP without the CBM illustrating fluorescence at the base and throughout the root hairs; (C, E and F) etiolated cotyledons showing fluorescence from AtGH9C1-GFP with the CBM in stomata; (D) etiolated cotyledons showing fluorescence from AtGH9C1-GFP without the CBM in the vascular traces and through-out all cells. (F) GFP overlay on the surface of etiolated cotyledons shown in E (40X); (F inset), Optical cross section through an open stomate showing GFP signal within the stomate cavity. (TIF) [file pone.0049363.s003.tif]

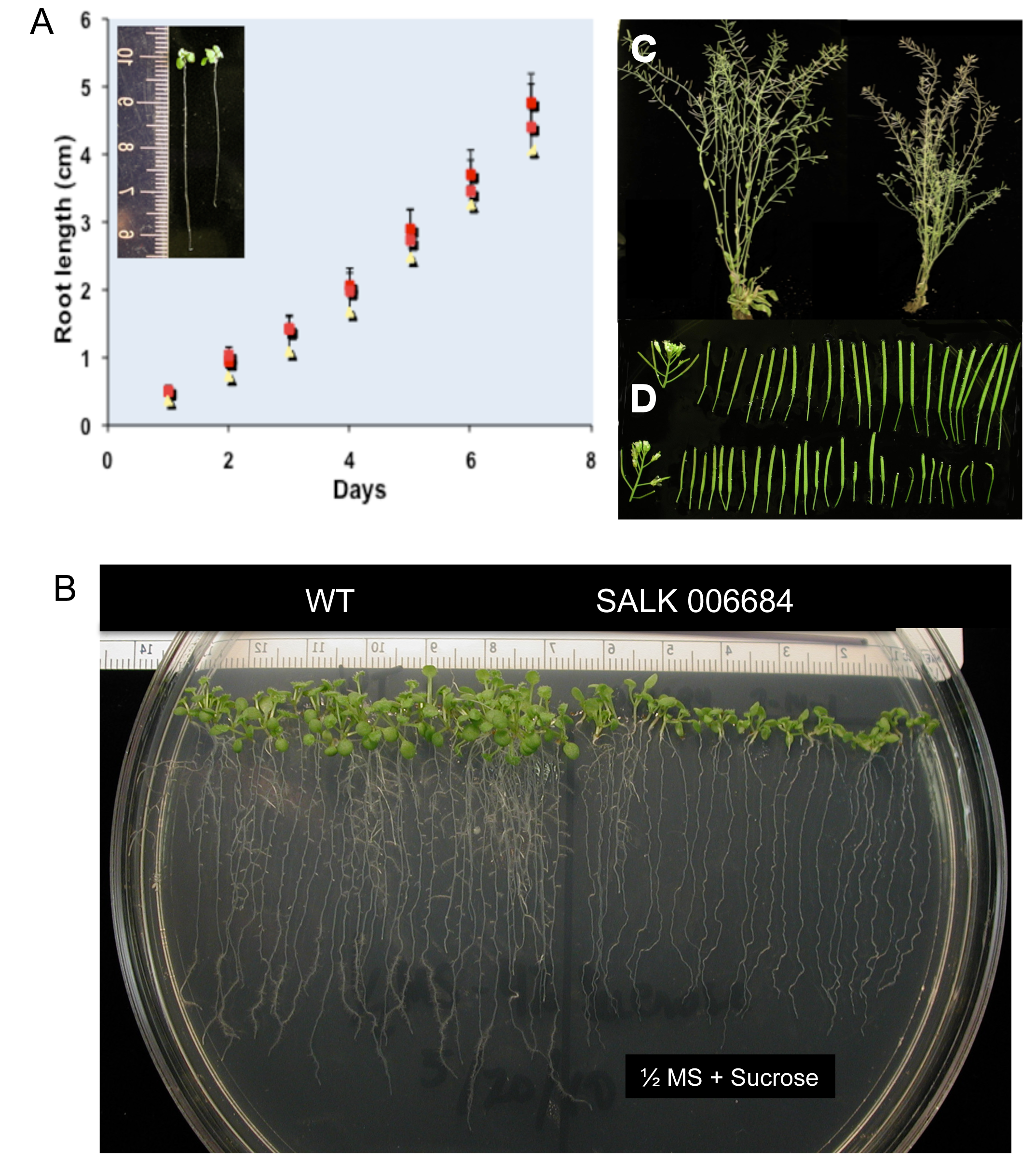

Supplement: Figure S4 — Wild type versus SALK 006684 Characterization. (A) Root length of SALK 006684 (yellow triangles) and wild type Columbia (red square) were measured daily during a week of growth in control agar plates, starting a day after transfer to 20°C; inset illustrate the difference between both lines, 5 days after transfer to 20°C. (B) Wild type and SALK 006684 growing in media supplemented with sucrose. Note the presence of abundant root hairs in wild type compared to seedlings of SALK 006684. (C) Plant stature of wild type (left) and SALK 006684 (right) growing in soil with regular watering for 6 weeks. (D) Inflorescence and siliques from the main stem of wild type (top) and SALK 006684 (bottom) (TIF) [file pone.0049363.s004.tif]

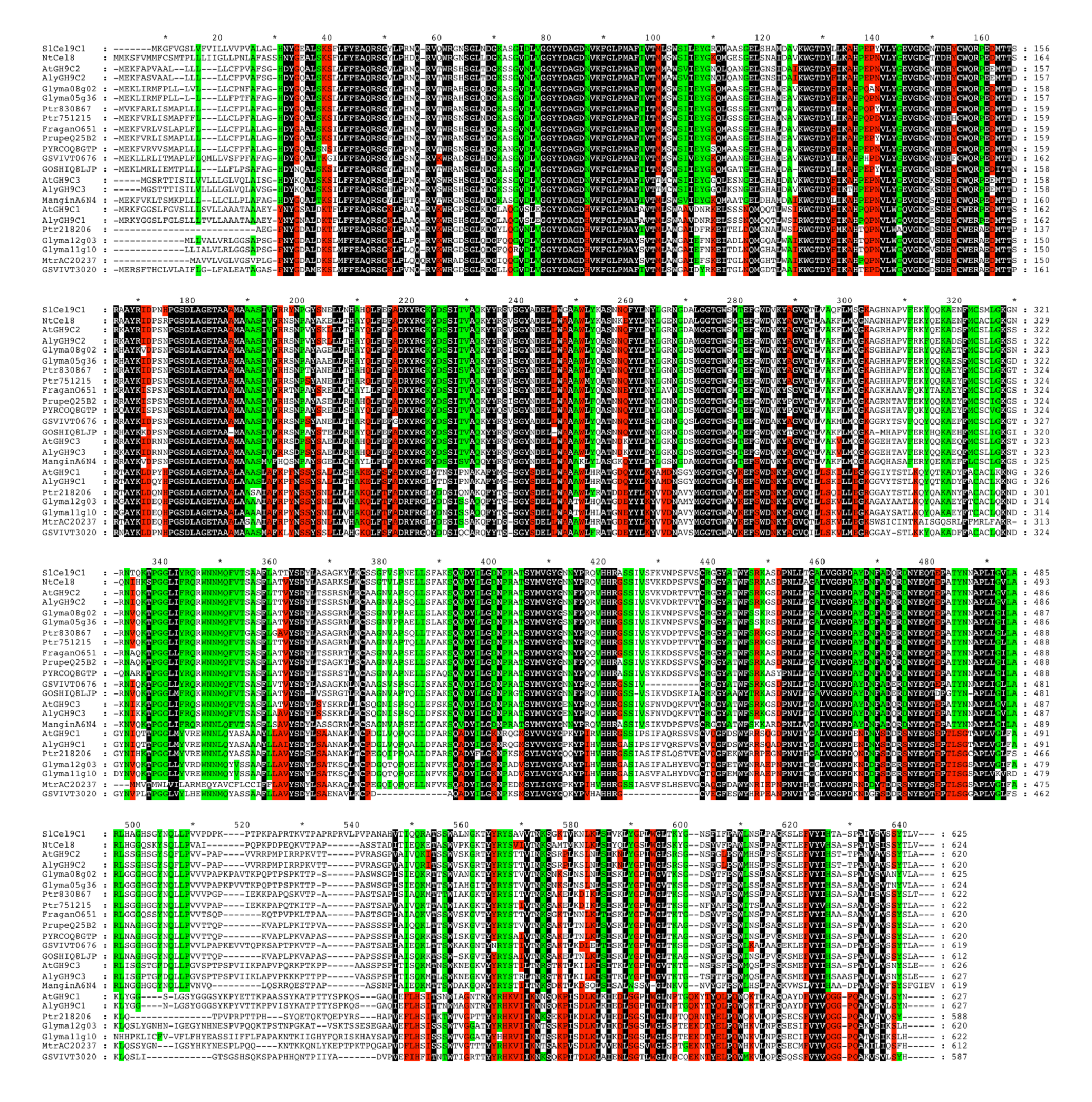

Supplement: Figure S5 — Amino acid alignment of dicots GH9 with a C-terminal extension (Blosum 62) illustrating the conserved amino acids in black, the unique amino acids of CLADE 1 in red and the unique amino acids of CLADE 2 in green. (TIF) [file pone.0049363.s005.tif]
